# Design of Dipeptite-Based Organogelators as Separators of Cationic Dye Cyristal Violet from Water

**DOI:** 10.3390/gels12040337

**Published:** 2026-04-17

**Authors:** Gülşen Kaya, Mehmet Çolak, Halil Hoşgören, Necmettin Pirinccioglu

**Affiliations:** 1 Department of Chemistry, Faculty of Science, Inonu University, 44280 Malatya, Turkey; 2 Department of Chemistry, Faculty of Science, Dicle University, 21280 Diyarbakir, Turkey

**Keywords:** environmental remediation, dye removing, pollutant removal, smart materials, PSOGs, crystal violet dye

## Abstract

The development of new advanced functional materials from low-molecular-weight gelators and their new potential applications have occupied a considerable place in research. The present study involves the design of dipeptide-based organogelators with enhanced hydrogen bonding network potentials and phase-selective capacities, possessing a minimum gelation concentration of 0.2–0.4% *w*/*v* in different fluids. Seven new dipeptide organogelators were prepared based on a one-step reaction from two-component salt forms, the combination of Nε-alkanoyl-L-lysine ethyl ester with N-alkanoyl-L-amino acids (L-alanine, L-leucine, and L-phenylalanine), with high yields of up to 90. All the gel materials were extremely stable at room temperature, having a shelf life of several months, and formed gels in pharmaceutical fluids such as ethyl palmitate, ethyl myristate, and ethyl laurate, 1,2-propanediol, and liquid paraffin (oils widely used in pharmaceutical formulations), which meet the criteria of biological materials delivery. Their gelation properties were evaluated by rheological measurements. A very significant breakthrough in the current study is that organogels remove the toxic dye, crystal violet (CV), from water in a phase-selective manner with an extremely low gelator concentration. The dye and gelators are successively recovered via ethanol precipitation after the completion of the phase extraction process. Molecular dynamic calculations provide evidence for the 3D structures of the gels.

## 1. Introduction

**Scheme 1:**
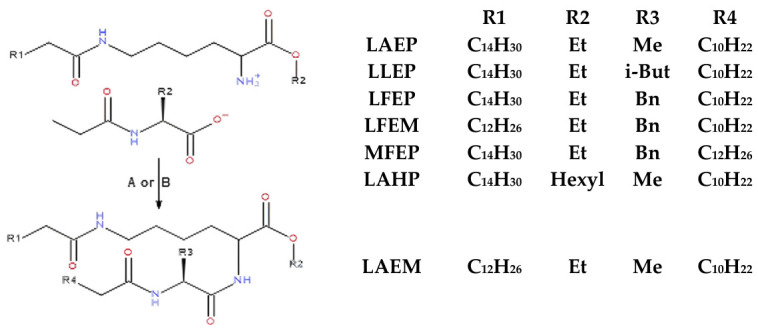
A. Coupling using EDC-HCl/HOBt reagents. B. Boric acid-catalyzed dehydration. The letter code represents the first letter of alkanoyl and alkyl groups in the peptides with the order of R4, R3, R2, and R1, where L stands for lauroyl, M for myristoyl, P for palmitoyl, E for ethyl, and H for hexyl. The second letter in the code corresponds to amino acid residues; A stands for alanine, L for leucine, and F for phenylalanine.

Low-molecular-weight gelators (LMWGs) possess many attractive properties [1]. They are easily synthesized and have tunable functionalities and low minimum gelation concentrations (MGCs). Therefore, they are considered attractive new soft materials [2] with a variety of applications in chemistry, materials, biology, food, cosmetics, and biomedicine, with an emphasis on different popular research areas [3,4,5,6,7,8,9], such as drug delivery [10,11,12,13,14,15], chemical sensing [16], and environmental processes [17]. Industrial developments produce massive wastes, which are consequently the main cause of environmental problems. In particular, the textile industries are the major contributors to water pollution, discharging non-biodegradable toxic dyes, threatening aquatic life, affecting freshwater ecology, and, consequently, human health [18,19,20,21,22]. Therefore, eliminating these life-threatening chemicals from aquatic life is an important goal in combating major environmental issues. There are several approaches employed in the removal of toxic dyes from the ecological biosphere in order to protect aquatic life. They include carbon black adsorption, chemicoagulation, sedimentation, and advanced oxidation procedures [23,24,25,26]. Among these techniques, adsorption is one of the choices since no harmful substance is formed. LMWGs are extensively employed in selective gelation and dye removal from water [27,28,29,30,31,32,33,34]. Although there are a large number of LMWGs designed with a wide range of chemical structures, the preparation of molecules to gelate specific solvents for a desired application is still challenging [35,36,37,38]. The key point is to control the gel properties, such as the ability to gelate different liquids, critical gel concentration (CGC), gel strength, and thermal stability, while preserving the main intermolecular interactions, which determine the supramolecular gel formation in a particular solvent and lead to fine-tuning between gel properties and desired applications [39,40,41,42,43,44,45,46,47,48,49,50]. This study involves the synthesis of seven dipeptide-based organogelators by two different coupling reactions from a two-component approach, boric acid-catalyzed dehydration and coupling by EDC-HCl/HOBt reagents (Scheme 1). One of the components is Nε-fatty acid acylated ethyl or hexyl L-lysine, and the other component is N-fatty acid acylated L-alanine, L-phenylalanine, or L-leucine. So, the molecules are rationally designed with minimal chemical modifications to have a large scale of properties to gelate a broad range of solvents such as lauric acid ethyl ester (LEE), myristic acid ethyl ester (MEE), palmitic acid ethyl ester (PEE), 1-decanol, 1,2-propanediol, liquid paraffin, and ethanol, which are widely used in the pharmaceutical and cosmetic industries. As a result, they form strong gels in these solvents with a wide range of properties for a specific application.

## 2. Results and Discussion

### 2.1. Synthesis of Organogelators

Hydrogen bonding, together with non-covalent interactions such as π–π stacking, plays a key role as the primary driving force behind the self-assembly of gelator molecules during gel network formation [51]. In this study, we have designed seven new gelators (Scheme 1) with inspiration from previously designed two-component gelator systems [45]. In this design, hydrogen bonding capacity between the gelators is increased by the addition of two acceptors (N-H) and two donors (C=O) groups compared to the former two-component structures. Moreover, apart from the existence of an optimum number of hydrogen bonding sites, the long lipophilic alkyl chains, different alkyl ester groups, and combinations of diverse amino acids provide functional diversity for the current dipeptide organogelators. The synthesis and applications of L-lysine-based LMWOGs have been widely reported [37,45,46,47,48]. Our dipeptide precursors, L-lysine-based two-component systems, were prepared according to the procedure described in the literature [45].

Here, we described the synthesis of seven dipeptides with a variety of alkanoyl chain lengths both on L-lysine and on the amino acid partner. Besides one of the organogelators (LAHP), ethyl was replaced with n-hexyl in the ester function of L-lysine. The synthesis of organogelators based on the coupling of two components by boric acid catalysis and EDC-HCl/HOBt afforded dipeptides with high yields (78–89%). These organogelators with a simple structural change on the two-component precursor systems involving amidation of basic and acidic components exhibited a significant reduction in the MGC in a wide range of pharmaceutical fluids. Boric acid-catalyzed coupling relies on the amidation of two-component salt forms. The release of water during the coupling process is trapped by using the Dean–Stark apparatus. Although this method has a simple synthetic procedure and limited chemicals, it involves some disadvantages. The reaction is extremely slow and frequently requires additional chromatographic purification steps. The second method is based on the employment of EDC-HCl/HOBt as coupling reagents. The coupling reactions have a short reaction time and a high reaction yield. On the other hand, the method bears some disadvantages, such as the use of expensive and non-green reagents. The acylation of lysine proceeds in a chemoselective manner, favoring the acylation of the Nε amino group over the Nδ one, thus not requiring the protection/deprotection procedure. As a consequence, from the green chemistry point of view, using cheap and environmentally friendly biodegradable materials makes boric acid-catalyzed coupling the choice for the synthesis of these organogelators.

### 2.2. Gelation and Gel Properties

**Figure 1:**
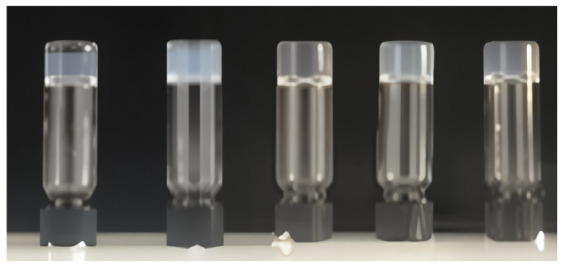
From left to right, the images of MFEP, LFEM, LLEP, LAHP, and LFEP gels in 1,2-propanediol prepared in their minimum gel concentrations (mg/mL). The pictures are produced by using the vial inversion method.

The gelation test was carried out following the procedure reported in the literature [52]. A gelator (1 mg) was dissolved in 1 mL of an organic solvent. The mixture was heated to a temperature of 20 °C below the boiling point of the solvent until the gelator was completely dissolved. The resulting solution was then kept at room temperature under static conditions. After approximately 15 min, gel formation was checked using the vial inversion method (Figure 1). If gelation did not occur, an additional 1 mg of gelator was added, and the procedure was repeated until gelation was observed. The concentration at which gelation occurred was recorded as the MGC (mg/mL). If 1 mg of gelator could not be dissolved in 1 mL of the specified solvent, it was reported as insoluble in that solvent. On the other hand, if the gelator was soluble in more than 10% (*v*/*v*) of the specified solvent, it was classified as soluble in that solvent. The results indicate that the gelators, except LAEP and LAEM, have excellent ability to gelate various pharmaceutical liquids such as LEE, MEE, and PEE, as well as liquid paraffin and 1,2-propanediol (Table 1). The reason why LAEP and LAEM did not gelate the mentioned solvents may be associated with the fact that they both possess an alanine residue, as a part of the dipeptide, resulting in notable insolubility in these solvents and consequently not showing the ability to gelate them. LAEP forms an opaque gel in 1,2-propanediol, and LAEM forms a transparent gel in liquid paraffin (Table 1). This is likely due to the low lipophilic character of the alanine residue since LAHP has an alanine residue, but the ethyl ester group is replaced with hexyl, as compared to LAEP, which gelates LEE, MEE, PEE, and liquid paraffin. On the other hand, all the gelators are soluble in ethanol and 1-decanol, except MFEP, which is not soluble in ethanol, and therefore, they did not demonstrate the ability to gelate these solvents. So, it appears that the hydrophilic–hydrophobic balance between the gelator and the solvent is possibly a required criterion in the gelation process of the current molecules.

**Figure 2:**
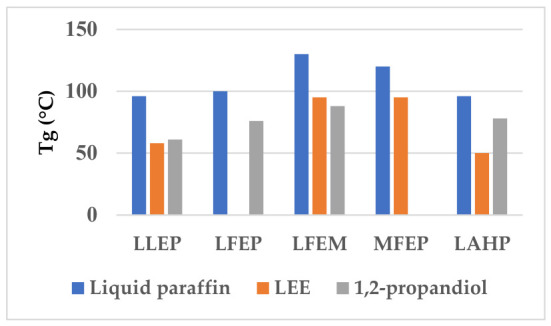
The concentration-dependent plateau Tg values of the gels in liquid paraffin, LEE, and 1,2-propanediol.

The measurements of gel–sol transition temperatures (Tg), namely the melting points, provide information about the thermal stability of the gels, as commonly reported for low-molecular-weight gel systems in the literature [53,54]. The concentration-dependent melting points for LLEP, LFEP, LFEM, MFEP, and LAHP in liquid paraffin, LEE, and 1,2-propanediol are shown in Figure 2. The measurements for LFEP in LEE and MFEP in 1,2-propanediol could not be obtained because of insolubility or weak gelation, respectively. The data demonstrate that, as anticipated, the melting points (Tg) of the gelators increase with increasing gelator concentration, which is consistent with previously reported supramolecular gel systems [55,56]. This behavior indicates that increasing gelator concentration enhances the density of the self-assembled network, leading to a higher number of gelator molecules per unit volume participating in the thermal transition process. Notably, gels containing phenylalanine moieties (LFEM, MFEP, and LFEP) in liquid paraffin, LEE, and 1,2-propanediol exhibit higher transition (melting) temperatures compared to the other systems, which is in good agreement with previous studies reporting the significant role of amino acid-derived and aromatic functionalities in strengthening gel networks via non-covalent interactions such as hydrogen bonding and π–π stacking [57,58]. Hence, it appears that the presence of the aromatic ring plays a central role in the self-assembly of these gelator molecules, most likely through π–π stacking interactions. A second notable observation is that gels LLEP, LFEP, and LAHP, which possess identical alkanoyl chains (lauroyl and palmitoyl), exhibit closely related Tg values in liquid paraffin and partially in 1,2-propanediol and LEE. This highlights the significant contribution of hydrophobic interactions to the gelation process. The final remark derived from the concentration-dependent melting point data is that the thermal stability of the gels strongly depends on the polarity of the solvent (Figure 2). Overall, all gels exhibit higher melting points (LLEP: 98 °C, LEEP: 100 °C, LFEM: 125 °C, MFEP: 120 °C, and LAHP: 98 °C) in liquid paraffin, the least polar of the solvents used. Although a direct comparison for LEE and 1,2-propanediol is limited due to missing Tg values for certain systems (Figure 2), it can still be inferred that lower polarity solvents favor stronger self-assembly, most likely by enhancing hydrogen-bonding interactions, particularly evident for LFEM across the examined solvent systems.

### 2.3. Nature of Intermolecular Interactions in Gels

**Figure 3:**
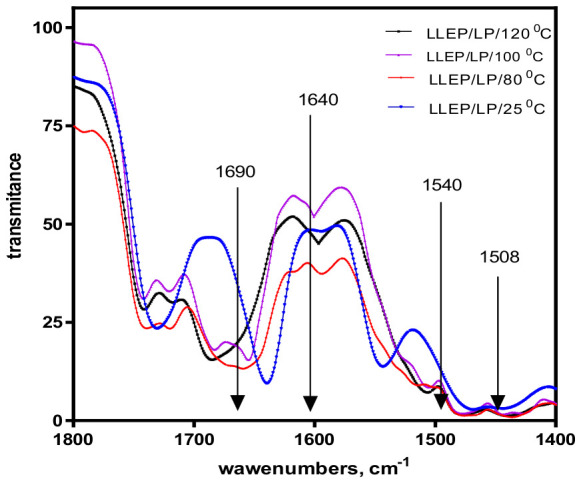
The temperature-dependent (25–120 °C) FTIR spectrum of LLEP gel in liquid paraffin. The variation in the wave number of amide I and amide II bands.

**Figure 4:**
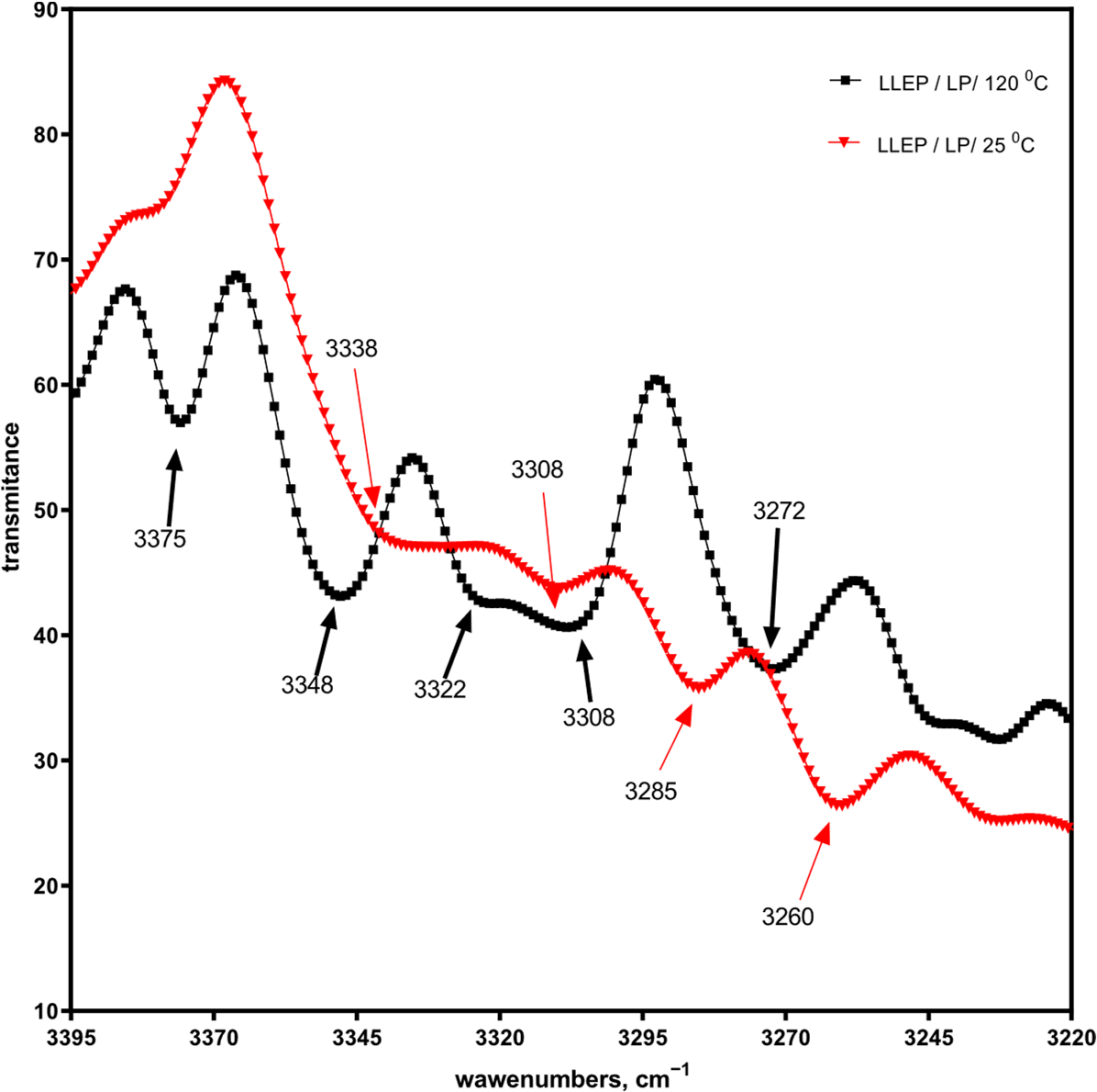
The temperature-dependent FTIR spectrum of the LLEP gel in liquid paraffin. The wave number variation in the N-H stretching band.

Gelation is a phenomenon where molecules form supramolecular aggregates via intermolecular interactions to form three-dimensional fibrous assemblies [59]. FT-IR analysis is a useful tool to highlight intermolecular hydrogen bonding in the formation of gels. These intermolecular hydrogen bonding interactions and the resulting three-dimensional fibrous network are expected to play a crucial role in the dye adsorption performance by providing interconnected voids, active binding sites, and a porous microenvironment for dye uptake. The examination of the temperature-dependent FT-IR spectra of LLEP gels in liquid paraffin provides important insights into the nature of the hydrogen-bond network in the gels. It is evident that the ester function is not involved in intermolecular hydrogen bonding during gel formation since the carbonyl peak at 1700–1780 cm^−1^ does not undergo any shift with increasing temperature. On the other hand, the shift in the amide I band from 1640 cm^−1^ to 1690 cm^−1^ and that in the amide II band from 1540 cm^−1^ to 1508 cm^−1^ are indicative of the main role of amide bonds in the formation of the gel network via hydrogen bonding (Figure 3). Moreover, the temperature-dependent shift in N–H stretching to higher wavenumbers supports N–H···O=C hydrogen bonding in the gel network (Figure 4).

**Figure 5:**
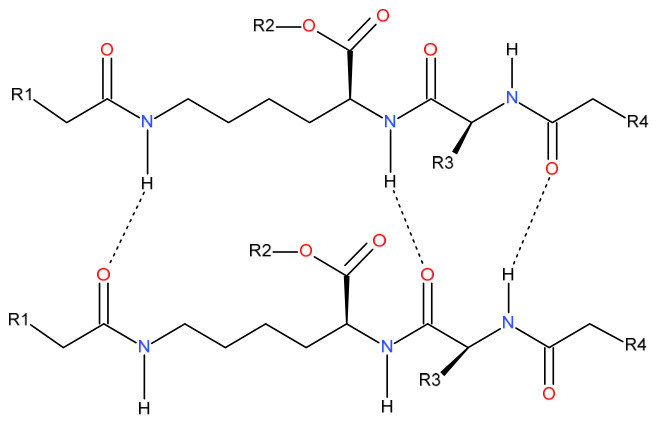
A proposed model for the self-aggregation of the gelators.

Moreover, the shift in the C–H stretching band of LLEP from 2966 cm^−1^ in the gel state to 2929 cm^−1^ in chloroform solution demonstrates intermolecular interactions between the alkanoyl groups. This shift was previously observed and attributed to strong aggregate formation and a consequent decrease in the fluidity of the hydrophobic chains through van der Waals interactions [60]. The involvement of intermolecular interactions was also confirmed by the dilution of the xerogel solid form of the LLEP gelator in chloroform (10^−3^ mol/dm^3^). The spectrum of LLEP in the solid state shows an amide I band at 1634 cm^−1^ and C–H stretching bands at 2929 cm^−1^. These stretching bands correspond to hydrogen-bonded N–H and C=O groups in the amide linker and van der Waals interactions between hydrophobic alkanoyl chains, respectively. When LLEP is diluted in chloroform, the peaks shift to higher wavenumbers (1659 cm^−1^ and 2966 cm^−1^, respectively). On the other hand, the peak corresponding to amide II at 1541 cm^−1^ in the solid state shifts to 1518 cm^−1^ in the solution state. Thus, a 2D model can be proposed for the self-aggregation of the gelators based on the observations obtained from FT-IR analyses (Figure 5).

### 2.4. Electron Microscopy

**Figure 6:**
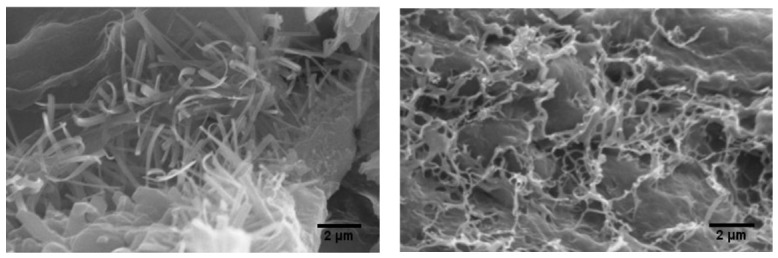

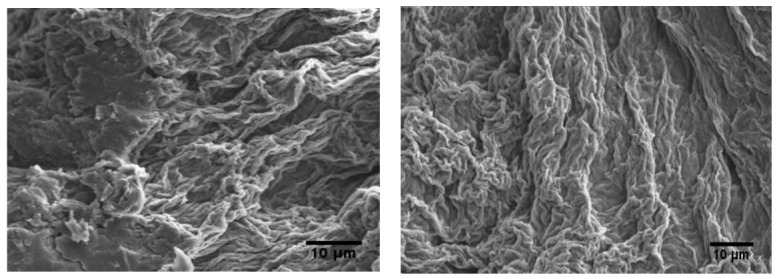
SEM images of xerogels. (**left top**) LLEP (12 mg) in 1 mL of toluene. (**right top**) LFEM (12 mg) in 1 mL of toluene. (**left bottom**) MFEP (12 mg) in 1 mL of toluene. (**right bottom**) LAHP (12 mg) in 1 mL of toluene.

Scanning electron microscope images of xerogel forms of LLEP, LFEM, MFEP, and LAHP in toluene were obtained to observe the nature of the microstructures and morphologies of these gels (Figure 6). They demonstrate that the amino acid and alkyl groups of the ester function clearly influence the structure of gelator morphology. The LFEM organogel, composed of Nε-myristoyllysine ethyl ester and N-lauroylphenylalanine, forms loose and relatively soft fibrils in toluene (Figure 6). On the other hand, MFEP, different from LFEM by only two carbons on Nε of lysine, composed of Nε-palmitoyllysine ethyl ester and N-myristoylphenylalanine, displays rather strict fibrils (Figure 6). LAHP, composed of Nε-palmitoyllysine hexyl ester and N-laurylalanine, has a similar morphology to MFEP (Figure 6). On the other hand, LLEP gel, composed of Nε-palmitoyllysine ethyl ester and N-lauroylleucine, exhibits a ribbon fiber skeleton instead of a fibrillary structure (Figure 6). So, we may speculate that the hexyl ester group in LAHP and the higher lipophilic character of alanoyl chains in MFEP further strengthen the nonbonding interaction in the gelator molecules because of their more fibrillary structures. This situation is clearly seen in their SEM images. The ribbon structure prevented the speedy adsorbate structure of LLEP.

### 2.5. Rheological Properties

**Figure 7:**
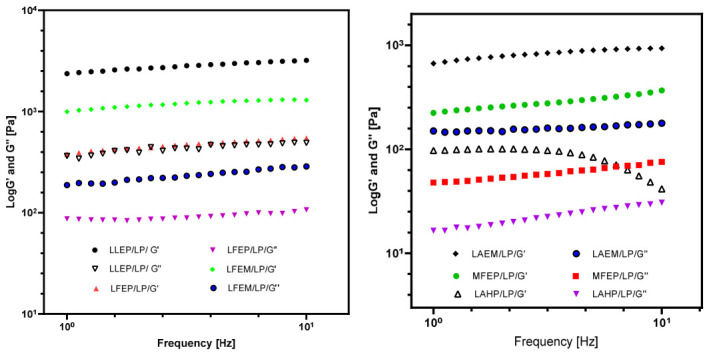
Double logarithmic graph of gels with a concentration of 4 mg mL^−1^ in liquid paraffin. The log storage modulus and log loss modulus are plotted as a function of log oscillating frequency measurement at 25 °C. Each curve represents the mean value of three tests.

The measurement of an oscillatory amplitude sweep produces two parameters: the storage modulus (G′), a measure of the ability of the deformed material to restore its original geometry, and the loss modulus (G″), a measure of the tendency of a material to flow under stress. Gels, considered as viscoelastic materials, have a higher value of G′ than G″. This means that the elastic behavior of the system is dominant. As a consequence, this brings about a lower value of G″/G′, which corresponds to the loss factor defined as tanδ = G″/G′ and is also called the stress amplitude sweep experiment. So, each gel owns a particular tanδ based on its strength or rigidity to an oscillating stress [54,61,62]. The results of the oscillation stress sweep experiment for the gels in liquid paraffin (Table 2) and in toluene demonstrate that the storage modulus (G′) is higher than the loss modulus (G″) in all the organogels (Table 3). They also show that both moduli are quite independent of frequency, and hence, all the gels have a typical viscoelastic behavior with good tolerance to external forces [37]. So, it means that the gels in the tested fluids form an elastic gel structure, and therefore, they can be categorized as members of the strong gel class. Gels have viscoelastic properties and therefore display resistance to flow under applied stress. The dynamical rheological behavior in a large spectrum of frequencies is characterized by double logarithmic graphs of oscillating frequency measurement. Data indicate that the organogel network has a long enough lifetime to consider the systems as gels (Figure 7).

**Figure 8:**
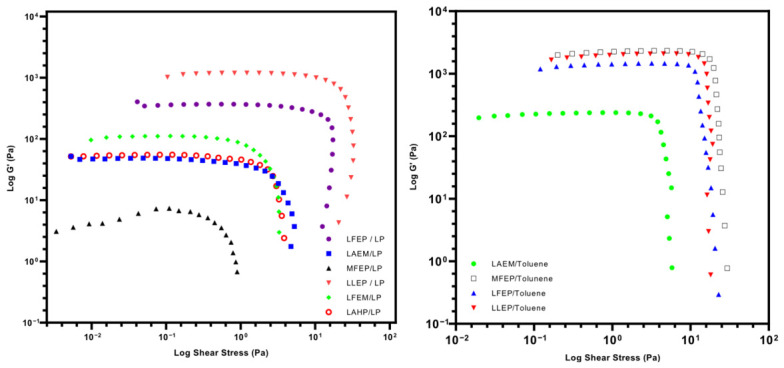
The storage modulus of the gelators as a function of shear stress in liquid paraffin (**left**) with a concentration of 4 mg mL^−1^ and in toluene (**right**) with a concentration of 12 mg mL^−1^. Each curve represents the mean value of three tests.

One disadvantage of supramolecular gels is that they have relatively low mechanical strengths, which arise from weak non-covalent interactions. This imposes a restriction with respect to their applications in physical manipulation. The G′ values of LLEP, LFEM, MFEP, and LAHP in toluene gels at the end point were found to be about 2030 Pa, 1440 Pa, 211 Pa, and 2250 Pa, respectively, and correspondingly, their yield stresses were 9.45 Pa, 6.75 Pa, 3.13 Pa, and 10.5 Pa (Table 3). These suggest that the minor change in a gelator structure could significantly influence the rheological properties of a gel (Figure 8). On the other hand, the G′ values of the same gelators in liquid paraffin at the end point were 1000 Pa, 305 Pa, 36.9 Pa, and 6.53 Pa, respectively, and correspondingly, their yield stresses were 10.3 Pa, 6.7 Pa, 1.19 Pa, and 0.211 Pa. So, this implies that solvents have a significant influence on the rheological properties of gels.

### 2.6. Dye Adsorption into Self-Assembled Gels

Dye removal experiments were performed using crystal violet (CV) as a model organic dye in a biphasic toluene–water system. Typically, 2.0 mL of an aqueous CV solution with a known concentration (see Supporting Information for the calibration curve) was placed in a vial, and 0.5 mL of toluene was carefully added to form a biphasic system (water:toluene = 4:1, *v*/*v*). To ensure complete gelation of the organic phase, a predetermined amount of dipeptide-based gelator was added, and the mixture was briefly heated. After cooling to room temperature, gel formation occurred in the toluene phase, during which the transfer of CV from the aqueous phase into the gelled organic phase was observed.

At predetermined time intervals, aliquots were withdrawn from the aqueous phase, and the residual dye concentration was determined by UV–Vis spectroscopy at the maximum absorption wavelength of CV. Since CV is not soluble in toluene, control (blank) experiments were not required. Dye concentrations were calculated using a previously established calibration curve (provided in the Supporting Information). The adsorption capacity and removal efficiency were evaluated based on the time-dependent decrease in absorbance. It was confirmed that dye removal was primarily associated with the gel phase rather than the solvent. All experiments were performed at least in duplicate, and average values are reported.

Importantly, upon completion of the phase extraction–gelation process, the dye-loaded gelator separated as a floating phase on top of the aqueous layer. This dye-containing upper phase was carefully collected using a spatula. The collected material was then treated with ethanol; the gelator was successfully recovered via ethanol-induced precipitation, while the toxic CV dye was transferred and dissolved in the ethanol phase. These results clearly demonstrate the recyclability of the system.

**Figure 9:**
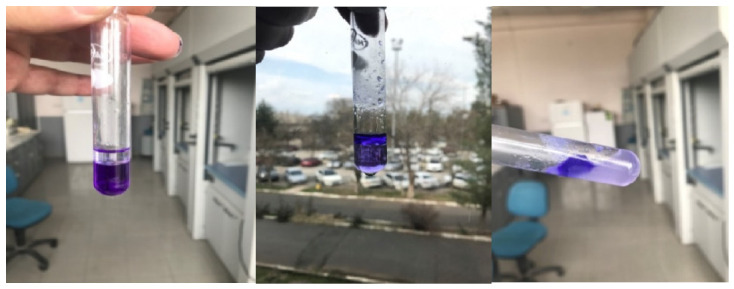
Representative steps in the adsorption of CV from aqueous solution to LAHP gel in toluene. (**left**) The tube has two layers with a ratio of 4:1 (*v*/*v*); the bottom is water (2 mL) containing 1 × 10^−6^ M of the dye, and the upper is toluene (0.5 mL) containing 6.0 mg of LAHP. (**middle**) The migration of the dye from the aqueous phase into the toluene layer after gentle heating and shaking. (**right**) The dye was completely removed from the water layer at room temperature within 10 min.

The amount of adsorbed dye over time was determined by UV–Vis spectroscopy (Table 4), enabling the evaluation of the time-dependent adsorption behavior of the studied dipeptides. The results demonstrate that MFEP and LAHP gels exhibit a strong capability for removing CV dye (Figure 9). Within 2 h, the dye was almost quantitatively removed from the aqueous phase (>95%), highlighting the high efficiency of these gelators. Although this rapid dye removal process requires heating, it offers a promising approach for the treatment of non-biodegradable dyes in textile wastewater.

Among the studied compounds, MFEP and LAHP gels acted as highly efficient adsorbents, removing nearly all of the dye (>90%) within 2 h. It is proposed that ester functionalities provide favorable binding sites for dye molecules, most likely through electrostatic interactions. The higher adsorption capacity of LAHP supports this hypothesis, as the hexyl chain in its ester moiety may further enhance complex formation via π/CH interactions between the gel and the dye.

In contrast, the exceptional performance of MFEP is more difficult to rationalize; however, the presence of benzene rings appears to play a significant role. This is also supported by the significantly higher adsorption capacity of LFEM compared to LLEP (81.9% vs. 38.2%, Table 3). Two possible mechanisms may explain this behavior: (i) a shift in binding sites from ester groups (as in LAHP) toward aromatic regions, and (ii) stabilization of the gel network through benzene rings, which strengthens the hydrogen-bonded framework and enhances dye–gel interactions.

### 2.7. Computational Modeling

**Figure 10:**
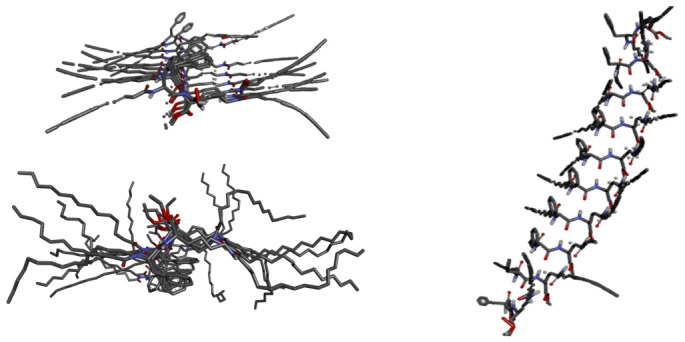
The representation of the structures of the decamer of MFEP in toluene obtained from MD calculations. Hydrogens are omitted for clarity. (**upper left** and **right**) Different perspectives on the average structures. (**lower left**) The structure with the largest population obtained from the cluster analysis of molecular dynamic trajectories.

**Figure 11:**
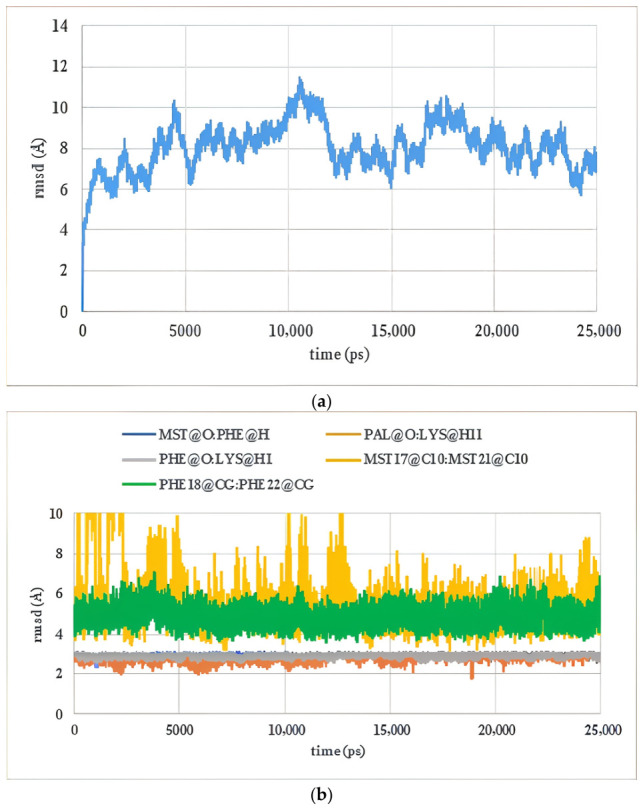
(**a**) RMSD of the decamer during the MS simulations, relative to its starting coordinates. (**b**) Bond distances during MD calculations in the decamer for a period of 25 ns. The mean values for each bond is found as 2.93 ± 0.08 for MST@O:PHE@H, 2.79 ± 0.18 for PHE@O:LYS@H1, 2.92 ± 0.07 for PAL@O:LYS@H11, 5.62 ± 1.61 for MST17@C10:MST21@C10, and 4.94 ± 0.42 for PHE18@CG:PHE22@CG.

**Figure 12:**
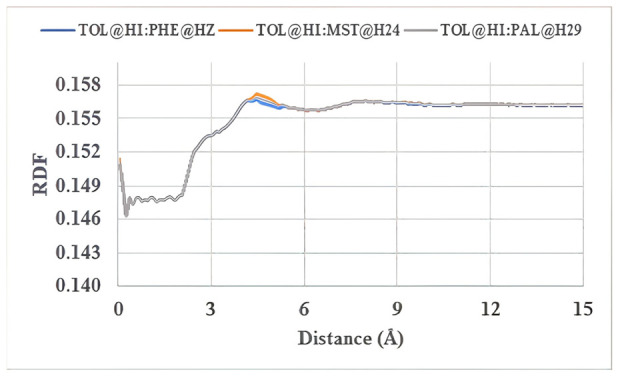
Radial distribution functions (RDFs) for the interactions of benzylic hydrogens of toluene with para-hydrogen of the phenyl ring in phenylalanine and the hydrogens attached to the 3rd carbon of myristoyl and palmitoyl from the carbonyl.

The structure of the decamer obtained from molecular dynamic simulation in a toluene solvent box for a period of 25 ns is shown in Figure 10. The frame of the decamer is slightly bent, posing an α-helix-like structure. The average structure shows that the decamer is relatively uniform during the dynamic calculations, and slight conformational changes are only seen in the alkanoyls and the phenyl rings in two edge chains (Figure 10 and Figure 11). This is supported by the maintenance of intermolecular interactions involving largely the hydrogen bond network, the π-π stacking between the phenyl in phenylalanine, and the hydrophobic interactions between the carbon chain of lysine and partially between the alkanoyl groups during the molecular dynamic simulations (Figure 12). The benzene rings also interact with the lysine carbon chain via π/CH interactions. The stacking pattern of the benzene rings is interestingly quite similar to that of nucleotide rings in DNA and RNA (Figure 10).

It is evident that the π-π stacking plays a crucial role in the conservation of the hydrogen bonds and hence the strength of the gels. These are consistent with the experimental results where the phenylalanine-based gels have relatively higher gel–sol transition melting points. It is also apparent that the alkanoyl site chains in the gels are compact and do not undergo substantial conformation changes during MD calculations (Figure 12) since there is no significant distance change between the fifth carbons from the carbonyl carbon of two neighboring myristoyl chains in E and F chains (Figure 11). The other outcome consistent with the experimental results obtained from FT-IR measurements is that the ester functions are not involved in the hydrogen bonding during molecular dynamic calculations.

The calculation of radial distribution functions (RDFs) [63] from molecular dynamic trajectories provides significant information regarding the probability of finding a particle at a distance from another particle. So, it is possible to find the contact distances for certain points. The distances between specific atoms in the solvent (benzylic hydrogens) and their potential interactions with certain atoms in the gelator (the hydrogen in the phenyl ring of phenylalanine and the hydrogen attached to the third carbon of alkanoyls) are calculated (Figure 12). They show the density distribution of the solvent around atoms in the decamer. This means that it is more likely that the solvent molecules would be in contact with the gelator at around 4.5 Å.

## 3. Conclusions

Seven lysine-based dipeptides have been synthesized with high yields in one step from two-component precursor systems, which involve simple, readily, and naturally available starting materials. They bear four alternative sites, two alkanoyl chains, the alkanoyl in the ester function, and an amino acid, which provide opportunities to alter their gelation capacity. The solvents also pose an alternative for the change in the gelation capacity of the gelators. These alternative modifications make the gelators potential competitors to the polymeric gelators. Their excellent abilities to gelate water-immiscible solvents, such as liquid paraffin, toluene, and other pharmaceutical fluids, pose many advantages for various applications.

The FT-IR analyses show that the peptide functions are involved in the hydrogen bondings to maintain the 3D structures of the gels, while the ester function does not take part in the intermolecular interaction of this kind, which is consistent with the computational modeling. The gel–sol transition melting points demonstrate that all the gelators form thermally stable gels in the tested solvents, particularly those bearing phenylalanine residue, possibly due to the strong interaction between the benzene rings through π-π stacking, which is confirmed by molecular dynamic calculations.

The investigation of the ability of the gels to clean very common dyes from the wastewater contaminated by textile industries demonstrates that the gels, particularly LAHP and MFEP, and partially LFEM, have a powerful capacity to remove crystal violet dye from water by the phase-selective gelation of the gelators in the water–toluene system. The dye and gelators can be recovered by ethanol precipitation after the completion of the phase extraction process. It appears that the modifications in the alkanoyl of the ester, the site chains of amino acids, and the length of the alkanoyl groups significantly influence the capacity of the gels to remove the dye from water. These organogels may be considered potential drug carrier materials in future studies since they have biodegradable and biocompatible features, as well as a moderate physical and chemical profile.

## 4. Materials and Methods

### 4.1. Materials and Instruments

Reagent-grade chemicals were used in all the reactions without further purification, unless otherwise mentioned. They were purchased from Sigma-Aldrich (Merck KGaA, St. Louis, MO, USA) suppliers. Since the substances used in reactions are sensitive to air and moisture, glass materials and solvents are dried, and all the reactions are carried out in a highly pure argon or nitrogen atmosphere. LEE, MEE, and NƐ-palmitoyl-L-lysine hexyl ester were prepared according to the literature procedures [52,53]. Melting point measurements were performed with a Gallenkamp Model device with open-ended capillary tubes. Results are given without error correction. Elemental analyses (C, H, and N) were accomplished using the Carlo Erba 1108 Elemental Analyzer (Carlo Erba Instruments, Milan, Italy). Results are given within 0.3% deviation. Mass spectra were taken with the Shimadzu LCMS-8040 (Shimadzu Corporation, Kyoto, Japan) instrument. Specific rotation angles were measured with a Perkin-Elmer 341 model polarimeter device at 20 °C in a 1 mL volume and a 1 dm long polarimeter cell. UV-vis spectroscopy was carried out on a VARIAN UV1010M211 (Varian Inc., Palo Alto, CA, USA) spectrophotometer. The reaction course was monitored by TLC-cards (F254). Silica gel (Merck, 0.040–0.063 mm) was used in flash chromatography. A Bruker Avance 400 MHz NMR spectrometer (Bruker Corporation, Billerica, MA, USA) was employed to record ^1^H NMR (400 MHz) and ^1^3C NMR (100 MHz) spectra. The residual solvent protons were used as an internal reference, and signals were recorded in ppm (δ). Fourier transform infrared spectroscopy (FTIR) with a Mattson 1000 ATI UNICAM (Unicam Ltd., Cambridge, UK) machine and a Specac GS20730 model (Specac Ltd., Orpington, Kent, UK) heating apparatus was used to record IR spectra. Samples are prepared in KBr pellets. The solution and solid-state samples of LLEP were obtained in non-gelling solvent chloroform and compressed KBr pellets, respectively, to show intermolecular driving forces between gelator molecules. An FEI Quanta 250 FEG (FEI Company, Hillsboro, OR, USA) field emission scanning microscope was used to record SEM images. An Anton Paar MCR 301 rheometer (Anton Paar GmbH, Graz, Austria) was employed to study rheological properties.

### 4.2. Synthesis

#### 4.2.1. General Synthesis of Dipeptides: Preparation of Two-Component Precursor Systems

The target precursors and their salt forms were prepared according to the literature method [54]. In this study, three different NƐ-alkanoyl-L-lysine alkyl esters, basic components, five different N-alkanoyl-L-amino acids, and acidic components were used as precursors.

#### 4.2.2. Coupling of NƐ-Alkanoyl-L-Lysine Alkyl Esters and N-Alkanoyl-L-Amino Acids Catalyzed by Boric Acid

Nε-Alkanoyl-L-lysine alkyl esters (1 mmol) in ether (10 mL) were added to the N-alkanoyl-L-amino acids (1 mmol) in methanol (10 mL). The mixture was stirred at room temperature for 30 min before being stored at 4 °C in the refrigerator for 1 day. The mixture of solvents was evaporated. The resultant precipitate was washed with cold ether and dried in a vacuum to give a two-component precursor salt [37]. The two-component precursor salt and H_3_BO_3_ (10 mg) were added to xylene (100 mL) in a 250 mL round-bottom flask. The Dean–Stark apparatus was attached to the bottle and boiled under reflux at 145 °C for 24 h. The mixture was cooled to room temperature; the light yellow, semi-solid material was obtained and filtered through a water jet pump and washed thoroughly with cold hexane. The solid material was purified by column chromatography on silica gel with EtOAc: chloroform (4:1) solvent system. The analytically pure product was recrystallized as a white solid from CHCl_3_.

#### 4.2.3. Coupling of NƐ-Alkanoyl-L-Lysine Alkyl Esters and N-Alkanoyl-L-Amino Acids by EDC-HCl/HOBt

N-Alkanoyl-L-amino acid (2 mmol) was dissolved in dichloromethane (40 mL) in an ice bath at 0 °C. At once, 384 mg (2 mmol) of EDC-HCl and 326 mg (2 mmol) of HOBt were added to the mixture. After stirring in an ice bath for 20 min, the solution of NƐ-alkanoyl-L-lysine ethyl or hexyl ester (2 mmol) in dichloromethane (30 mL) was added dropwise via a pressure-controlled funnel. The mixture was stirred at 0 °C for six hours and then overnight at room temperature. Dichloromethane was removed, and the reaction mixture was dissolved in chloroform. The organic phase was washed with 10% citric acid (6 × 10 mL). The organic layer was dried on Na_2_SO_4_ and then filtered and evaporated in a vacuum, producing a yellowish amorphous solid, which was crystallized from methanol to give a white solid. The analytically pure product was obtained by recrystallization of the solid from one of the following solvents: CHCl_3_, methanol, or ethanol.

#### 4.2.4. Coupling of NƐ-Palmitoyl-L-Lysine Ethyl Ester and N-Lauroyl-L-Alanine (LAEP)

The white solid was recrystallized from CHCl_3_: 78% (yield); Mp 148.2–149.7 °C; [α]D20 = −12.0 (CHCl_3_, c:1.0). The NMR spectrum was found to be consistent with the structure of LAEP. 1H NMR (400 MHz, CDCl_3_-d1): δ (ppm) 6.87 (d, NHC*HCO_2_C_2_H_5_, J = 8.0 Hz), 6.35 (d, NHC*HCH_3_, J = 8.0 Hz), 6.1 (bs, CONH), 4.17–4.52 (m, NHC*HCO_2_Et, COC*HCH^3^, 2H), 3.31–3.36 (OCH_2_CH_3_, 2H), 3.10–3.36 (m, LysNHCH_2_, 2H), 2.18–2.23 (dd, COCH_2_, 4H, J = 7.2 Hz), 1.63–1.89 (m, LysCH_2_, 6H), 1.51 (t, OCH_2_CH_3_, 3H, J = 6.8 Hz), 1.50 (d, CH_3_ C*H, 3H, J = 8.0 Hz), 1.26–1.42 (m, -CH_2_CH2-, 44H), and 0.89 (t, -CH_2_CH_3_, 6H, J = 7.2 Hz); 13C NMR (100 MHz, CDCl_3_-d1): δ (ppm) 173.93, 173.36, 172.39, 171.98, 61.47, 52.14, 48.85, 38.56, 36.69, 36.58, 31.93, 31.21, 29.71, 29.67, 29.64, 29.63, 29.56, 29.52, 29.42, 29.39, 29.37, 29.35, 29.30, 28.89, 25.92, 25.72, 22.69, 22.09, 18.22, 14.17, and 14.12; IR (KBr pellet, cm^−1^): 1538 (CONH amid II), 1648 (CONH amid-I), 1753(COCH_2_CH_3_), 2921, 2851(C-H), and 3318(N-H). Chemical analysis calculated for C_39_H_75_N_3_O_5_ C 70.32; H 11.34; N 6.32; found: C 70.53, H 11.32, N 6.30. LC-MS (ES) calculated for C_39_H_75_N_3_O_5_ (M)+ 666,02, found: 666.7.

#### 4.2.5. Coupling of NƐ-Palmitoyl-L-Lysine Ethyl Ester and N-Lauroyl-L-Leucine (LLEP)

A yellowish solid crystallized from methanol was obtained: 89% (yield); Mp 103–105 °C; [α]D20: +1.3 (CHCl_3_, c:1.0). ^1^H NMR (400 MHz, CDCl_3_-d1): 6.75 (d, NHC*HCO2Et, 1H, J = 5.2 Hz), 6.15 [d, NHC*H(i-but), 1H, J = 5.2 Hz], 6.12 (bs, 1H, LysCONƐH), 4.43–4.51 (m, NHC*HCO_2_Et, 1H), NHC*H(i-but), 4.14–4.22 [m, -OCH_2_CH_3_, (2H),], 3.06–3.37 (dh, LysNHCH_2_ (2H), 2.15–2.23 (m, COCH_2_, 4H), 1.46–1.90 [m, (CH_3_)_2_CH-, (1H), (CH_3_)_2_CHCH_2_-(2H), CH_3_CH_2_O-(3H), LysCH_2_ (6H)], 1.25–1.28 [(m, palCH_2_, (26H), (lau CH_2_, (18H)], and 0.94–0.96 [(dd, -CH(CH_3_)_2_]; ^13^C NMR (CDCl_3_-d1): δ (ppm) 173.73, 173.52, 172.21, 171.99, 61.44, 52.09, 51.77, 41.13, 38.96, 38.43, 36.89, 36.83, 36.63, 31.93, 31.92, 31.23, 29.72, 29.70, 29.68, 29.64, 29.57, 29.54, 29.46, 29.43, 29.38, 29.28, 29.06, 28.90, 28.90, 25.93, 25.87, 25.77, 24.77, 23.77, and 23.67; IR (KBr pellet, cm^−1^): 1659 (CONH amid-I), 1518 (CONH amid-II), 1732 (COEt), 2858, 3190, 3344 (N-H), and 3359. Chemical analysis calculated for C_42_H_81_N_3_O_5_: C 70.32; H 11.34; N 6.30; found: C 70.52, H 11.30, N 6.28. LC-MS (ES) calculated for C_43_H_81_N_3_O_5_ (M)+ 708.07, found: 709.

#### 4.2.6. Coupling of NƐ-Palmitoyl-L-Lysine Ethyl Ester and N-Lauroyl-L-Phenylalanine (LFEP)

The analytically pure product was obtained by crystallization of the white solid from CHCl_3_: 87% (yield); Mp 144.4–146.1 °C; [α]D20: +5.4 (CHCl_3_, c:1.0). ^1^H NMR (CDCl_3_-d1): δ (ppm) 7.21–7.30 (ArH, 5H), 6.42–6.44 [(CONℇH), (d, PhCH_2_C*HNH, (d,NHC*HCO_2_Et)], 4.46–4.47 [(m, (NHC*HCO_2_Et,1H), (Ph CH_2_C*HCO-,1H)], 4.15–4.20 (m, -OCH_2_CH_3_ 2H), 3.00–3.16 [(m, LysNHCH_2_, 2H), m,(PhCH_2_, 2H)], 1.60–1.64 (m, COCH_2_, 4H), 1.22–1.64 [ (bs (-CH_2_CH_2_-, 44H), (CH_3_ CH_2_O-, 3H), CH_2_Lys, 6H)], and 0.89 (t, CH_2_CH_3_, 6H, J = 6.8 Hz); ^13^C NMR (CDCl_3_-d1): δ (ppm) 174.09, 173.51, 171.67, 170.98, 136.48, 129.26, 128.61, 127.00, 61.48, 54.43, 52.18, 38.69, 37.99, 36.67, 36.56, 31.94, 31.34, 29.72, 29.68, 29.65, 29.57, 29.50, 29.44, 29.38, 29.18, 28.83, 25.96, 25.66, 22.71, 22.04, 14.18, and 14.14; IR (KBr pellet, cm^−1^): 1641 (CONH amid-I), 1543 (CONH amid II), 1736 (COCH_2_CH_3_), 2920, 2851, 3085 (ArH), and 3294 (N-H). Chemical analysis calculated for C_45_H_79_N_3_O_5_: C 72.82; H 10.74; N 5.68; found: C 73.07, H 10.75, N 5.70. LC-MS (ES) calculated for C_45_H_79_N_3_O_5_ (M)+ 742.07; found: 742.1.

#### 4.2.7. Coupling of NƐ-Myristoyl-L-Lysine Ethyl Ester and N-Lauroyl-L-Phenylalanine (LFEM)

The analytically pure product was obtained by crystallization of the white solid from CHCl_3_: 84% (yield); Mp.148.2–149.6 °C; [α]D20: +2.3 (CHCl_3_, c:1.0). ^1^H NMR (CDCl_3_-d1): δ (ppm) 7.20–7.29 m,(ArH, 5H), 6.59–6.60 (d, NHC*HCO_2_Et, J = 4.8 Hz], 6.18–6.19 (d, PhCH_2_C*HNH, J = 4.8 Hz], 5.91–5.93 [t, (CONH) J = 4.0 Hz], 4.65–4.68 [ (NHC*HCO_2_Et),1H J = 3.2 Hz], 4.44–4.48 [(m, (COC*HCH_2_Ph,1H) J = 4.8 Hz)], 4.12–4.20 (m, -OCH_2_CH_3_, 2H), 3.01–3.14 (dd, PhCH_2_, 2H)], 3.09–3.31 [(dh, LysNHCH_2_, 2H), 2.10–2.18 (m, -COCH_2_, 4H), 1.46–1.86 [(m, CH_3_CH_2_O-3H), (m, CH_2_Lys, 6H)], 1.25–1.30 [ (bs -(CH_2_CH_2_)5-, 40H), and 0.88 (t, CH_3_CH_2_-, 6H, J = 4.4 Hz); ^13^C NMR (CDCl_3_-d1): δ (ppm) 173.60, 173.39, 171.69, 170.97, 136.49, 129.25, 128.61, 127.00, 61.47, 54.34, 52.17, 38.56, 37.99, 36.86, 36.58, 31.93, 31.40, 29.71, 29.67, 29.64, 29.56, 29.49, 29.44, 29.41, 29.37, 29.18, 28.97, 25.90, 25.64, 22.70, 22.05, and 14.15; IR (KBr pellet, cm^−1^): 1544 (CONH amid-II), 1641 (CONH amid-I), 1751 (COEt), 1732, 2851, 2918, 3084 (ArH), and 3302 (N-H). Chemical analysis calculated for C_43_H_75_N_3_O_5_: C 72.31; H 10.58; N 5.88; found: C 72.51, H 10.63, N 5.92. LCMS (ES) calculated for C_43_H_75_N_3_O_5_ (M)+ 715.02; found: 715.05.

#### 4.2.8. Coupling of NƐ-Palmitoyl-L-Lysine Ethyl Ester and N-Myristoyl-L-Phenylalanine (MFEP)

The analytically pure product was obtained by crystallization of the white solid from CHCl_3_: 88% (yield); Mp 144.7–146.8 °C; [α]D20: −7.0 (CHCl_3_, c:1.0). ^1^H NMR (CDCl_3_-d1): δ (ppm) 7.20–7.29 m, (ArH, 5H), 6.58 (d, NHC*HCO_2_Et, J = 5.2 Hz], 6.17 (d, NHC*HCH_2_Ph, J = 5.2 Hz], 5.90 [t, (CONƐH) J = 3.6 Hz], 4.64–4.68 [q (NHC*HCO_2_Et),1H J = 4.8 Hz], 4.45–4.48 [(m, (COC*HCH_2_Ph,1H) J = 2.8 Hz)], 4.12–4.20 (m, -OCH_2_CH_3_, 2H), 3.01–3.14 (dd, PhCH_2_, 2H)], 3.09–3.31 [(dh, LysNHCH_2_, 2H)], 2.14–2.20 (m, -COCH_2_, 4H), 1.45–1.86 [(m, -OCH_2_CH_3_ 3H), (m, LysCH_2_, 6H)], 1.22–1.30 [(bs (-CH_2_CH_2_-, 48H), and 0.88 (t, -CH_2_CH_3_, 6H, J = 4.4 Hz); ^13^C NMR (CDCl_3_-d1): δ (ppm) 173.62, 173.40, 171.69, 170.96, 136.48, 129.26, 128.66, 128.58, 127.02, 61.48, 54.39, 54.30, 52.16, 38.56, 38.45, 38.11, 37.98, 37.86, 36.86, 36.58, 36.53, 31.94, 31.29, 29.72, 29.68, 29.50, 29.38, 29.18, 28.96, 26.07, 25.90, 25.64, 22.71, and 22.61; IR (KBr pellet, cm^−1^): 1543 (CONH amid-II), 1639 (CONH amid-I), 1731 (COCH2CH3), 2853 (C-H), 2922, 3082 (ArH), and 3288(N-H). Chemical analysis calculated for C_47_H_83_N_3_O_5_: C 73.28; H 10.87; N 5.45; found: C 73.52, H 10.88, N 5.47. LC-MS (ES) calculated for C_47_H_83_N_3_O_5_ (M)+ 770.12; found: 770.7.

#### 4.2.9. Coupling of NƐ-Palmitoyl-L-Lysine Hexyl Ester and N-Lauroyl-L-Alanine (LAHP)

The analytically pure product was obtained by recrystallization of the white solid from ethanol: 89% (yield); Mp 128.2–130.4 °C; [α]D20: −11.0 (CHCl_3_, c:1.0). ^1^H NMR (CDCl_3_-d1): δ (ppm) 6.92 (d, NHC*HCO_2_C_6_H_13_J = 8.0 Hz), 6.41 (d, NHC*HCH_3_ J = 7.2 Hz), 6.24 (LysNNH), 4.48–4.52 [(m, NHC*HCO_2_C_6_H_13_, NHC*HCH_3_, 2H)], 4.10–4.15 (m, OCH_2_CH_3_ 2H), 3.10–3.36 (m, LysNHCH_2_, 2H), 2.19–2.23 (m, COCH_2_, 4H), 1.15–1.88 [(bs, (-CH_2_CH_2_-, 50H), (d, NHC*HCH_3_, 3H, J = 8.0 Hz), (t, hex-OCH_2_CH_2_, 2H), (m, LysCH_2_, 6H)], and 0.89 (m,-CH_2_CH_3_, 9H); ^13^C NMR (CDCl_3_-d1): δ (ppm) 173.98, 173.38, 172.39, 172.06, 65.65, 52.14, 48.87, 38.57, 36.66, 36.57, 31.93, 31.77, 31.36, 31.25, 29.72, 29.68, 29.66, 29.58, 29.54, 29.44, 29.40, 29.38, 29.37, 29.31, 28.86, 28.47, 25.93, 25.73, 25.48, 22.71, 22.53, 22.09, 18.22, 14.14, and 14.01; IR (KBr pellet, cm^−1^): 1544 (CONH amid-II), 1639 (CONH amid-I), 1728 (COEt), 2852, 2922, and 3311 (N-H). Chemical analysis calculated for C_43_H_83_N_3_O_5_: C 71.51; H 11.58; N 5.83; found: C 71.73, H 11.63, N 5.85. LC-M(ES) calculated for C_43_H_83_N_3_O_5_ (M)+ 722.12; found: 722.7.

#### 4.2.10. Coupling of NƐ-Myristoyl-L-Lysine Ethyl Ester and N-Lauroyl-L-Alanine (LAEM)

The analytically pure product was obtained by recrystallization of the white solid from CHCl3 and then from ethanol: 85% (yield); Mp 147–149 °C; [α]D20: −4.6 (CHCl_3_, c:1.0). ^1^H NMR (CDCl_3_-d1): δ (ppm) 6.83 (d, NHC*HCO2Et, J = 7.2 Hz), 6.31 (d, NHC*HCH_3_, J = 7.20 Hz), 6.02 (t, CONH J = 6.0 Hz), 4.47–4.52 (m, NHC*HCO_2_Et, COC*HCH_3_, 2H), 4.15–4.22 (m,OCH_2_CH_3_ 2H), 3.09–3.36 (dh, LysNHCH_2_, 2H), 2.13–2.21 (m, COCH_2_, 4H), 1.60–1.90 (m, LysCH_2_, 6H), 1.47–1.52 (q, OCH_2_CH_3_, 3HJ = 7.2 Hz), 1.36–1.40 (d, NHC*HCH_3_, 3H), 1.25–1.28 (m, (-CH_2_CH_2_-, 40H), and 0.88 (t, -CH_2_CH_3_, 6H, J = 6.6 Hz); ^13^C NMR (CDCl_3_-d1): δ (ppm) 173.72, 173.28, 172.39, 172.00, 61.69, 61.47, 61.26, 52.15, 52.12, 48.83, 38.58, 38.45, 38.33, 36.82, 36.76, 36.59, 31.92, 31.43, 31.24, 31.04, 29.67, 29.64, 29.56, 29.53, 29.40, 29.30, 28.97, 28.79, 26.07, 25.88, 25.71, 25.53, and 22.80; IR (KBr pellet, cm^−1^): 1541 (CONH amid-II), 1645 (CONH amid-I), 1742 (COEt), 2851, 2920, and 3316 (N-H). Chemical analysis calculated for C_37_H_71_N_3_O_5_: C 69.67; H 11.21; N 6.58; found: C 69.88, H 11.24, N 6.61. LC-MS (ES) calculated for C_37_H_71_N_3_O_5_ (M)+; 637.97, found: 638.01.

### 4.3. Gelation Studies

#### 4.3.1. Determination of Minimum Gel Concentration (MGC)

The gelation test was performed according to the previously reported procedure [53]. The gelator concentration which generates gelation is noted as the minimum gelation concentration (MGC) (mg/mL). In cases where 1 mg of the gelator is not dissolved in 1 mL of the specified solvent, this is considered insoluble, and if the gelator is soluble in over 10% of the particular solvent, it is considered soluble in the specific solvent.

#### 4.3.2. Morphological Characterization by SEM

The nanostructures of the gels, which are in the form of a three-dimensional network [53], are observed in detail by the electron microscopy technique. The scanning electron microscopy (SEM) samples were prepared from gels in toluene by the freeze–drying method. The xerogel forms were prepared in 1 mL of toluene by taking 12 mg of gelator. Toluene was removed by freeze–drying for 24 h. The xerogel forms were only prepared for LLEP, LFEM, MFEP, and LAHP gelators, which were used in dye-removing experiments.

#### 4.3.3. Rheological Measurement

The viscoelastic properties of LLEP, LFEP, LFEM, MFEP, LAHP, and LAEM gels in liquid paraffin were measured using an Anton Paar MCR 301 rheometer with a 20 mm cone-plate with an adjustable temperature controlling system. The samples were prepared ahead of time and stored at a constant temperature (25 °C) overnight. For rheological measurements, a sufficient amount of gel was carefully taken using a spatula and placed on the rheometer plates prior to measurement. The width of the gap was 0.047 mm. The viscoelastic measurement of LAEP could not be taken in liquid paraffin due to its low solubility in this fluid. First, a stress sweep at a fixed frequency of 1 Hz allowed for determining the linear viscoelastic range (LVR) of the samples. In the stress sweep test, the storage modulus G′ and the loss modulus G″ were measured as a function of the stress amplitude in order to determine the linear viscoelastic range (LVR) and crossing over point (G′ = G′′) of the gels with (γ: 0.01–100%) strain at a constant frequency (10 rad s^−1^). Second, the frequency sweep test was obtained from 0.01 Hz to 10 Hz at a constant shear stress of 1.3 Pa that results in small strains. The frequency sweep test is frequently used to detect whether the sample is a true gel.

### 4.4. Modeling

The 2D structures were drawn by BIOVIA Draw 2019 [63]. The 3D structure of the decamer of LLEP was obtained from its 2D structure drawn by BIOVIA Draw 2019 [63]. The decamer was converted to a 3D structure by Discovery Studio 2021 Client [64] and minimized by employing Gaussian03 at the PM6 level [65]. The minimized structure was set apart into its monomers, and the coordinates of each monomer were saved in PDB format. Each monomer was assigned a chain, composed of four residues: myristoyl (MST), L-phenylalanine (PHE), ethyl ester of L-lysine (PLY), and palmitoyl (PAL). After the assignment of the residues in the monomer, 10 monomers were combined in a single file in a PDB format to gain the decamer with 10 chains, A–J. The molecular dynamic calculations were performed using Assisted Model Building with Energy Refinement (AMBER tools 18) [66]. The atomic charges of these residues, except L-leucine, were calculated by antechamber as implemented in AMBER. ff14SB [67] as implemented in AMBER was employed for the charge of L-leucine and also for topologies/parameters of all the residues. Antechamber was also used to derive atomic charges for toluene, which is used to solvate the decamer in a periodic box composed of 1255 molecules with an octagonal shape. Xleap as implemented in AMBER was used for producing topology and coordinate files for the molecular dynamic simulations. The minimization of the decamer in the periodic box of toluene was achieved in two steps. The first was carried out in 1000 steps with a constraint of 400 kcal mol^−1^ Å–2 on the decamer with two periods, 500 with the steepest descent method and 500 with the conjugate gradient method. All the atoms were allowed to move in the second step of minimization in 2500 steps, 1250 with the steepest descent method and 1250 with the conjugate gradient method. Heating was performed in the canonical ensemble for 200 ps, where the aggregates of the gelator were restrained with a force constant of 10 kcal mol^−1^ Å–2. The system was equilibrated for a period of 25 ns in a canonical ensemble at a temperature of 300 K and a pressure of 1 atm with a step size of 2 fs. A Langevin thermostat and barostat were employed to couple the temperature and pressure. The SHAKE algorithm was applied to constrain all bonds containing hydrogen atoms [68]. The non-bonded cutoff was kept at 10 Å, and long-range electrostatic interactions were treated using the particle-mesh Ewald (PME) [69] method with a fast Fourier transform grid with a spacing of approximately 0.1 nm. Sander as implemented in AMBER was employed for the minimization and molecular dynamic calculations. CPPTRAJ [70] module as implemented in AMBER was used to calculate RMSD changes, hydrogen-bond analyses, and radial distribution function (RDF) [71]. The graphical outcomes were represented by Excel or GraphPad Prism (9.3.0).

## Figures and Tables

**Table 1:** Minimum gel concentrations (mg/mL) of the gelators in pharmaceutical liquids.

	LAEP	LLEP	LFEP	LFEM	MFEP	LAHP	LAEM
LEE	2 (ns)	2 (tg)	2 (ns)	2 (tg)	6 (tl)	2 (tl)	2 (ns)
MEE	2 (ns)	2 (tg)	2 (tl)	2 (tg)	4 (tl)	2 (tl)	2 (ns)
PEE	4 (ns)	2 (tg)	2 (tl)	2 (tg)	4 (tl)	2 (tl)	4 (ns)
Liquid paraffin	4 (ns)	2 (tg)	2 (tg)	2 (tg)	4 (tg)	2 (tg)	2 (tg)
1,2-propanediol	4 (og)	2 (tg)	2 (tg)	4 (tl)	4 (tl)	4 (tl)	2 (ns)
1-decanol	10 (s)	10 (s)	10 (s)	10 (s)	10 (s)	10 (s)	10 (s)
Ethanol	10 (s)	10 (s)	10 (s)	10 (s)	2 (ns)	10 (s)	10 (s)

Description of gels: og: opaque gel; tg: transparent gel; tl: translucent gel; s: soluble; ns: not soluble.

**Table 2:** The oscillation stress sweep test for gelators in liquid paraffin at 25 °C at 0.0681% strain (G′ at the end value of LVR). In all the tests, the concentration of gelators was 4 mg mL^−1^ for those in liquid paraffin.

Gels	tanδ	G′ (Pa) ^a^	SS (Pa) ^b^	SS (Pa) ^c^	G′/G″ ^d^	G′ (Pa) ^e^
LLEP	0.176	1000	10.3	30	1000/236 = 4.23	208
LFEP	0.166	96.8	0.673	3.25	96.8/19.8 = 4.88	10.3
LFEM	0.155	305	6.70	17.00	305/59.8 = 5.10	56.30
MFEP	0.173	36.90	1.19	4.04	36.9/8.31 = 5.78	13.30
LAHP	0.488	6.53	0.211	0.90	6.53/1.31 = 2.05	6.53
LAEM	0.181	46	1.02	9.27	46/10.4 = 4.42	11

^a^: G′ (Pa) at the end point. ^b^: shear stress (Pa) at end point LVR. ^c^: shear stress (Pa) at crossing over point (COP). ^d^: ratio at the end point. ^e^: G′ (Pa)d at crossing over point.

**Table 3:** The oscillation stress sweep test for gelators in toluene at 25 °C at 0.0681% strain (G′ at the end value of LVR). In all the tests, the concentration of gelators was 12 mg mL^−1^ for those in toluene.

Gels	tanδ	G′ (Pa) ^a^	SS (Pa) ^b^	SS (Pa) ^c^	G′/G″ ^d^	G′ (Pa) ^e^
LLEP	0.069	2030	9.45	16.6	2030/187 = 10.85	463.5
LFEP	0.116	1440	6.75	12.8	1440/178 = 8.08	407
MFEP	0.068	211	3.13	4.65	211/27.4 = 7.70	58.05
LAHP	0.064	2250	10.50	21.40	2250/170 = 12.85	464

^a^: G′ (Pa) at the end point. ^b^: shear stress (Pa) at end point LVR. ^c^: shear stress (Pa) at crossing over point (COP). ^d^: ratio at the end point. ^e^: G′ (Pa)d at crossing over point.

**Table 4:** The percentage of CV dye absorbed from 2 mL (10^−6^ M) of dissolved solution by the LLEP, LFEM, MFEP, and LAHP gels prepared in toluene (the gelator concentration in toluene was taken to be 12 mg/mL).

Gelator	A ^a^	C ^b^	AdC ^c^	A ^d^	C ^e^	AdC ^f^
LLEP	0.881	0.5280	%14.2	0.653	0.3837	% 38.2
LFEM	0.593	0.3458	%44.5	0.239	0.1216	% 81.9
MFEP	0.095	0.0305	%97.1	0.086	0.0250	% 98
LAHP	0.110	0.0402	%97.5	0.065	0.0116	% 100

^a^ Absorbance after 2 h. ^b^ Concentration (mg·L^−1^) after 2 h. ^c^ Adsorption capacity after 2 h. ^d^ Absorbance after 4 h. ^e^ Concentration (mg·L^−1^) after 4 h. ^f^ Adsorption capacity after 4 h.

## Data Availability

The data are contained within the article.

## Supplementary Materials

The following supporting information can be downloaded at: https://www.mdpi.com/article/10.3390/gels12040337/s1.
