# Supplementary material for: Design of Dipeptite-Based Organogelators as Separators of Cationic Dye Cyristal Violet from Water
Source: Gels. 2026 Apr 17;12(4):337. doi: 10.3390/gels12040337 (PMC13116262; doi:10.3390/gels12040337)
Supplement: Supplementary file 1 [file gels-12-00337-s001.zip › gels-4244799-supplementary.pdf]

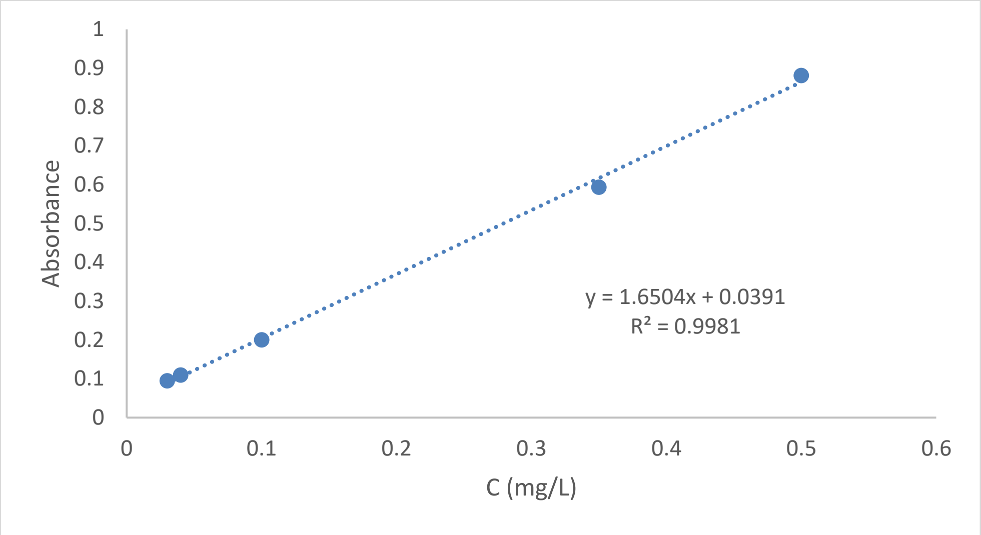

The calibration curve is established at 595 nm in the range of 0.03-0.5 mg/L CV.

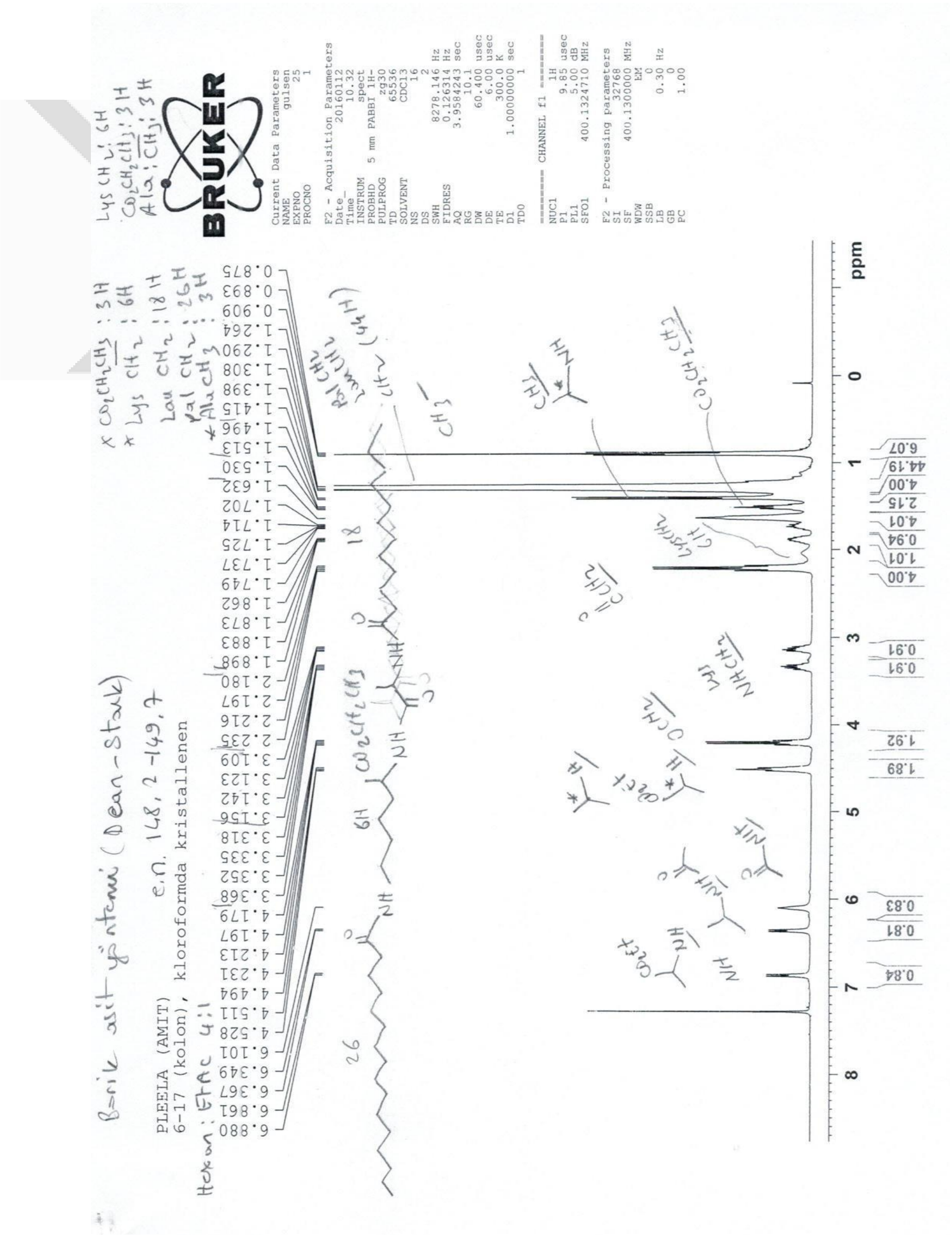

PLEELA (AMIT)
6-17 (kolon), kloroformda kristallenen

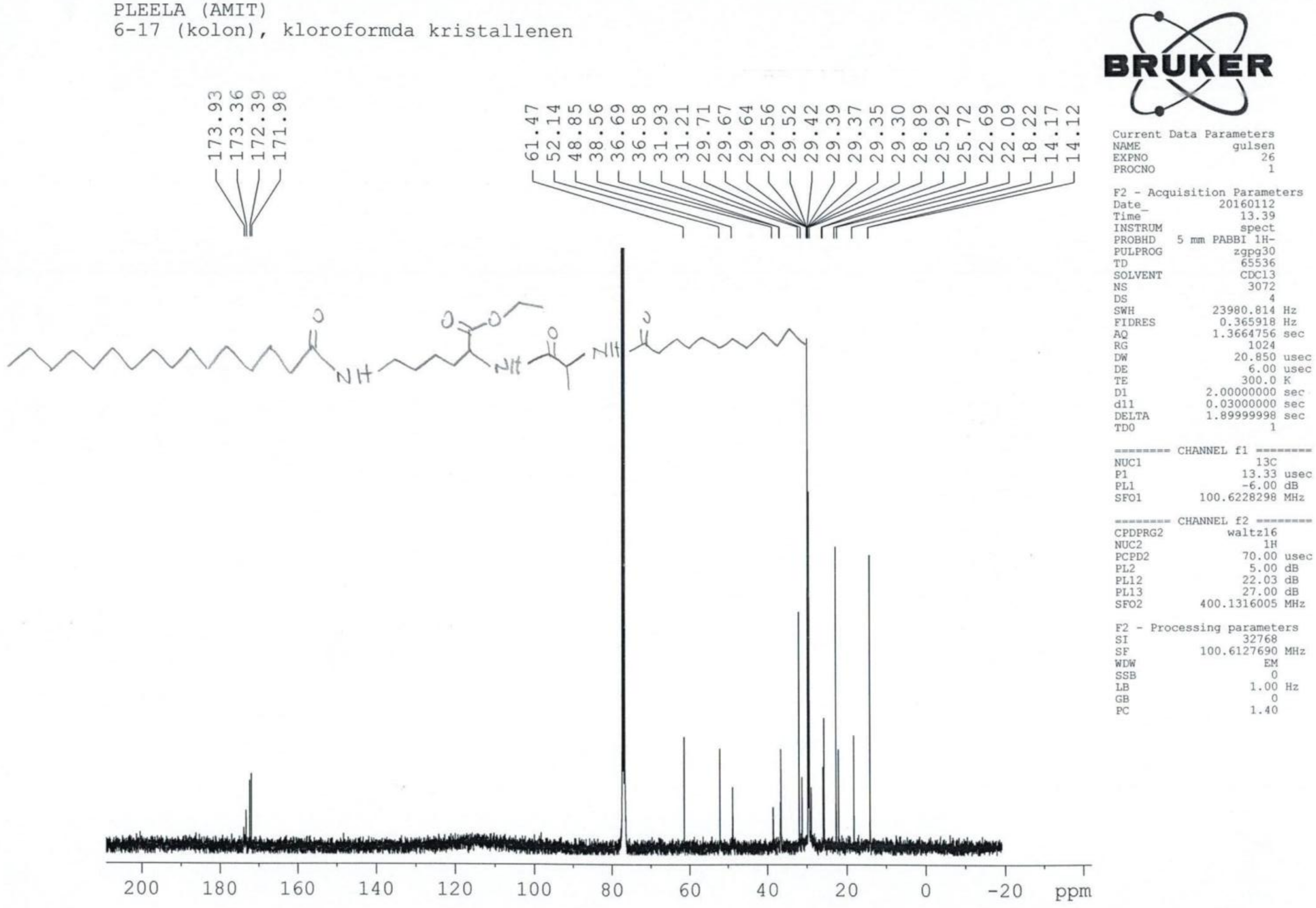

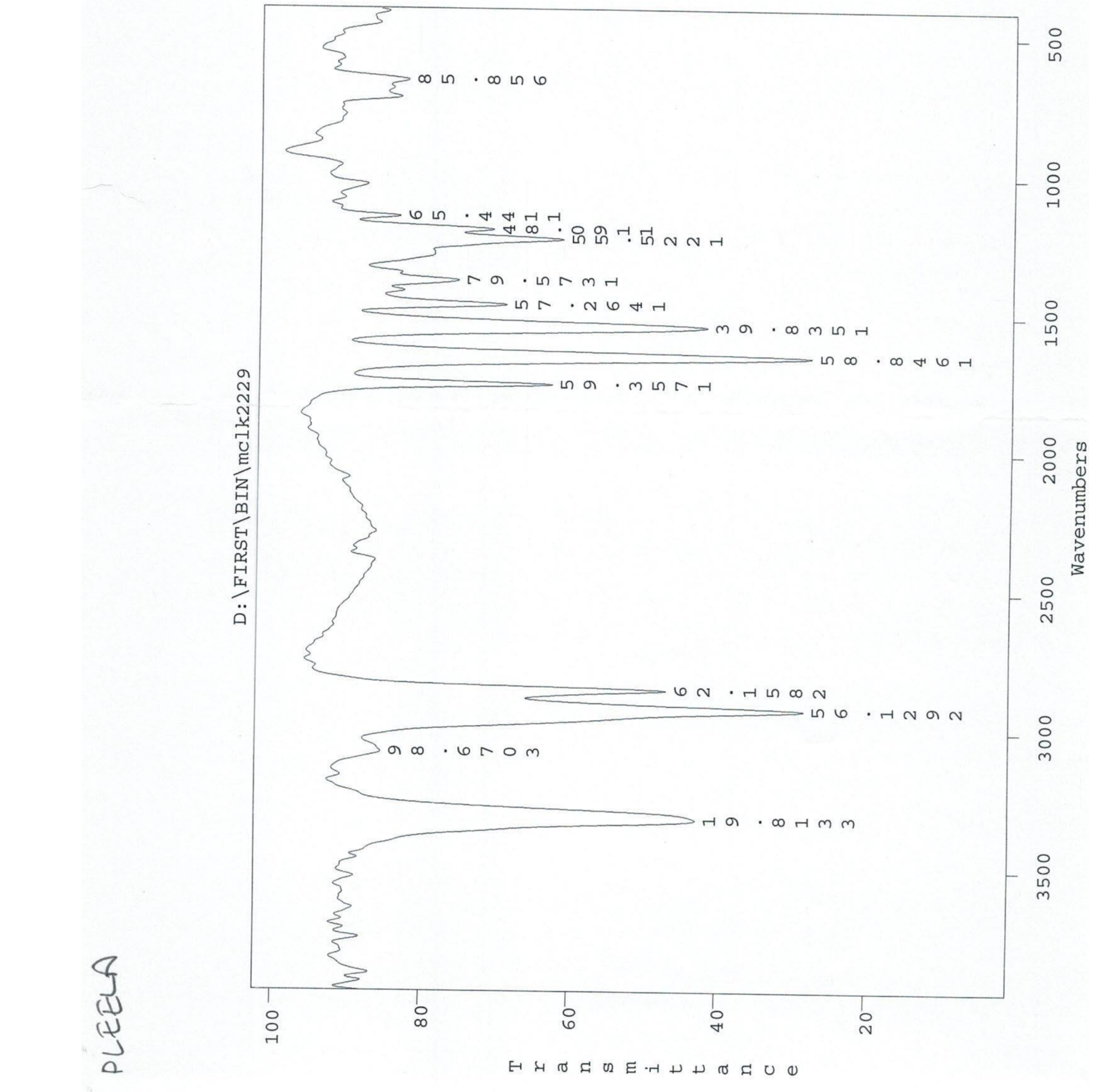

PLEELA
D:\FIRST\BIN\mclk2229
Transmittance
Wavenumbers
100
80
60
40
20
3500
3000
2500
2000
1500
1000
500
3318.91
3076.89
2851.26
1753.95
1648.85
1538.93
1462.75
1375.97
658.58

-

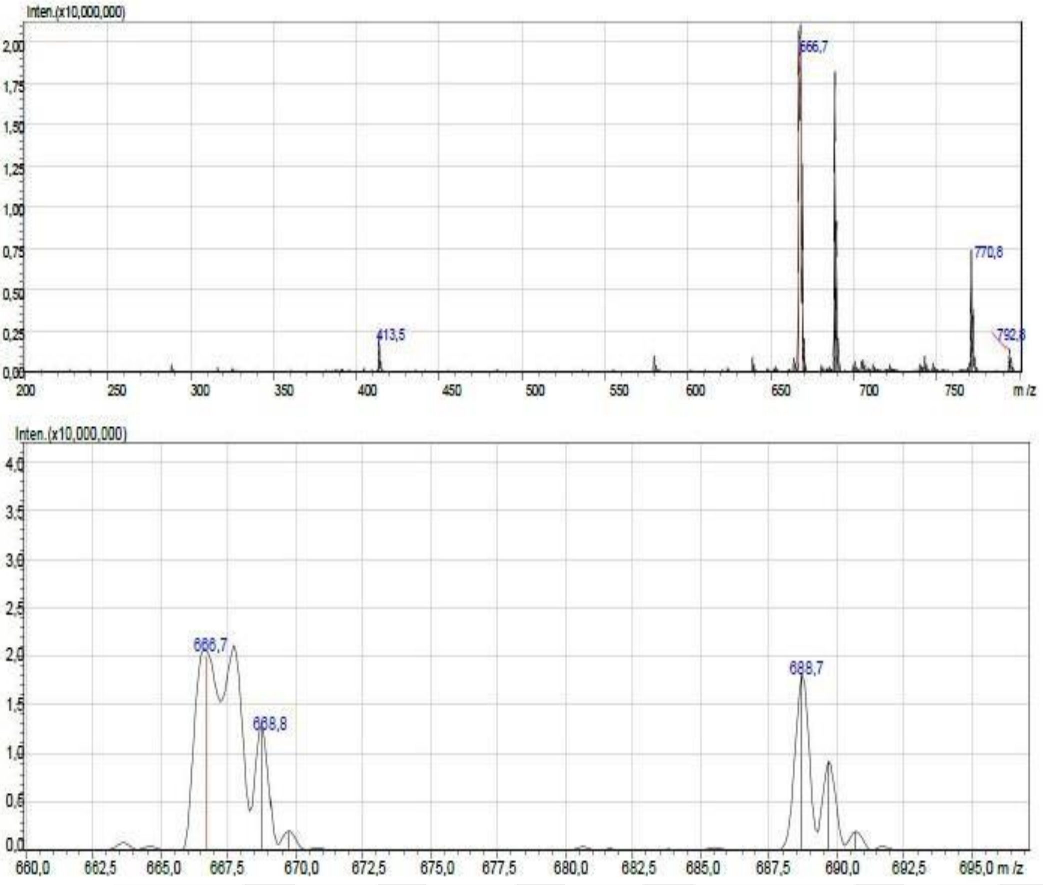
Inten.(x10,000,000)
666,7
770,8
413,5
792,8
m/z
Inten.(x10,000,000)
666,7
668,8
688,7
m/z

D2B JELATÖRÜNÜN NMR, IR VE MS SPEKTRUMLARI

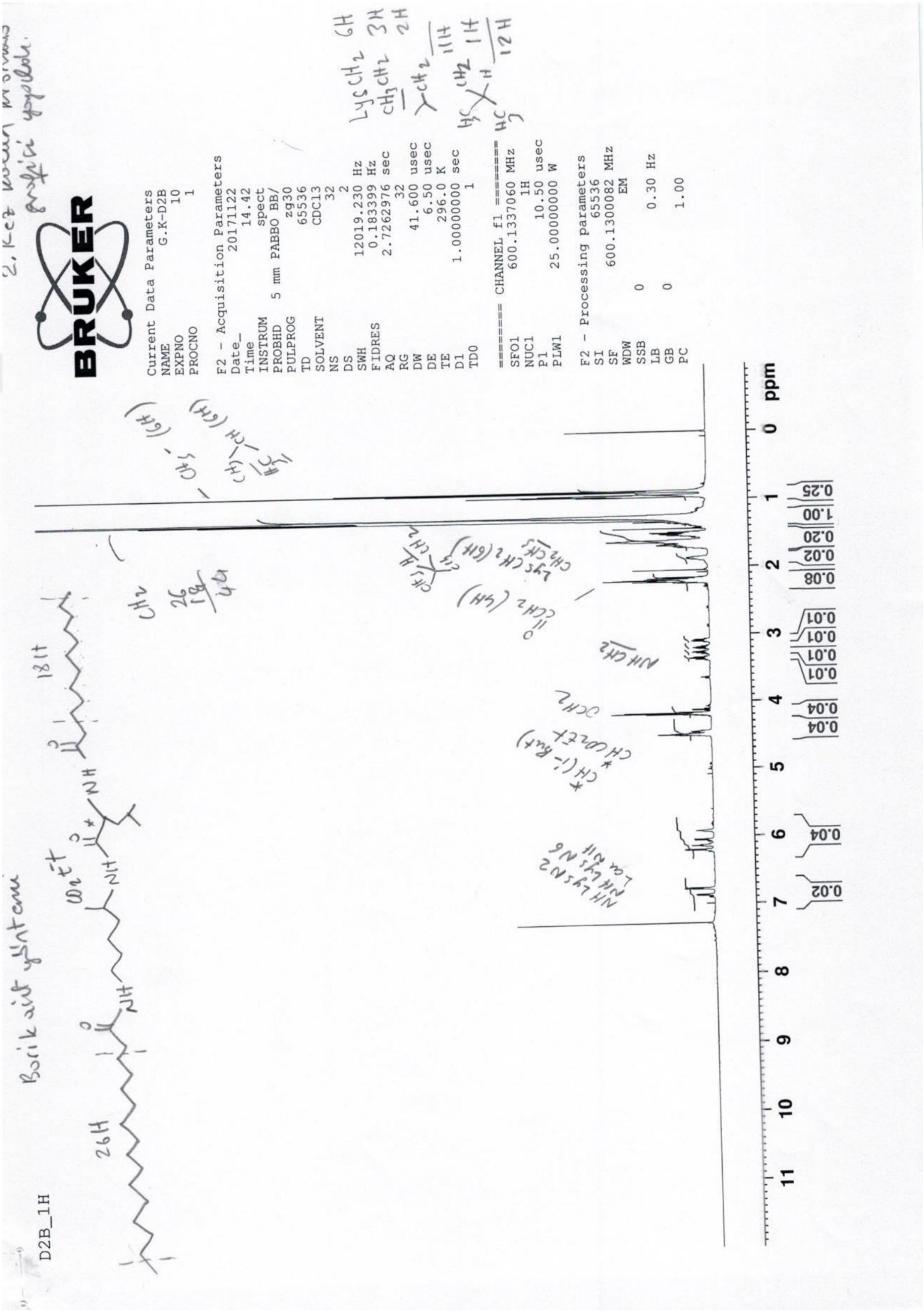

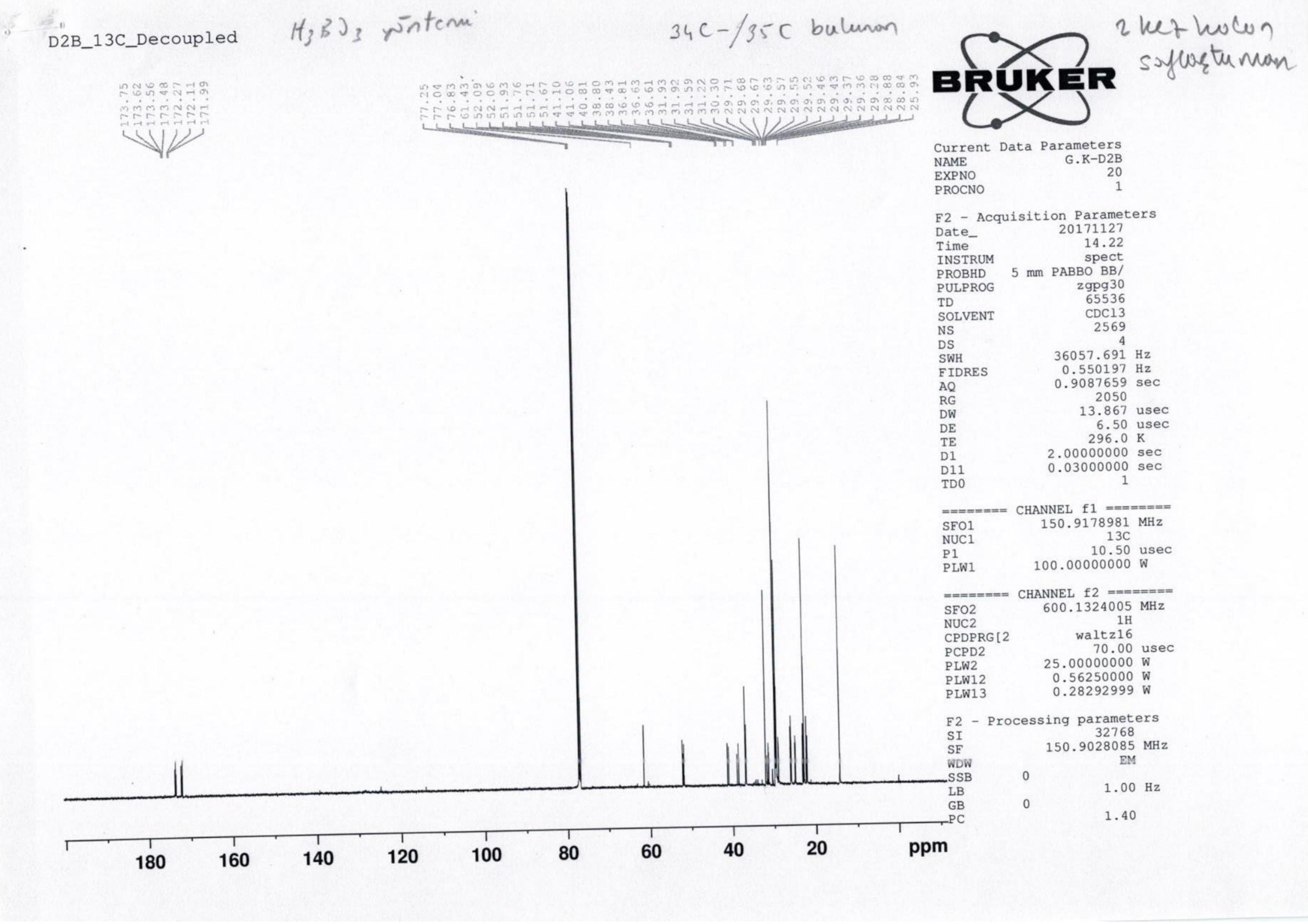
D2B_13C_Decoupled
173.75
173.62
173.56
173.48
172.27
172.11
171.99
77.25
77.04
76.83
61.43
52.09
52.06
51.93
51.76
51.71
51.67
41.10
41.06
40.81
38.80
38.43
36.81
36.63
36.61
31.93
31.92
31.59
31.22
30.30
29.71
29.68
29.67
29.63
29.57
29.55
29.52
29.46
29.43
29.37
29.36
29.28
28.88
28.84
25.93
BRUKER
Current Data Parameters
NAME G.K-D2B
EXPNO 20
PROCNO 1
F2 - Acquisition Parameters
Date_ 20171127
Time 14.22
INSTRUM spect
PROBHD 5 mm PABBO BB/
PULPROG zgpg30
TD 65536
SOLVENT CDCl3
NS 2569
DS 4
SWH 36057.691 Hz
FIDRES 0.550197 Hz
AQ 0.9087659 sec
RG 2050
DW 13.867 usec
DE 6.50 usec
TE 296.0 K
D1 2.00000000 sec
D11 0.03000000 sec
TD0 1
======== CHANNEL f1 ========
SFO1 150.9178981 MHz
NUC1 13C
P1 10.50 usec
PLW1 100.00000000 W
======== CHANNEL f2 ========
SFO2 600.1324005 MHz
NUC2 1H
CPDPRG[2 waltz16
PCPD2 70.00 usec
PLW2 25.00000000 W
PLW12 0.56250000 W
PLW13 0.28292999 W
F2 - Processing parameters
SI 32768
SF 150.9028085 MHz
WDW EM
SSB 0
LB 1.00 Hz
GB 0
PC 1.40
180
160
140
120
100
80
60
40
20
ppm

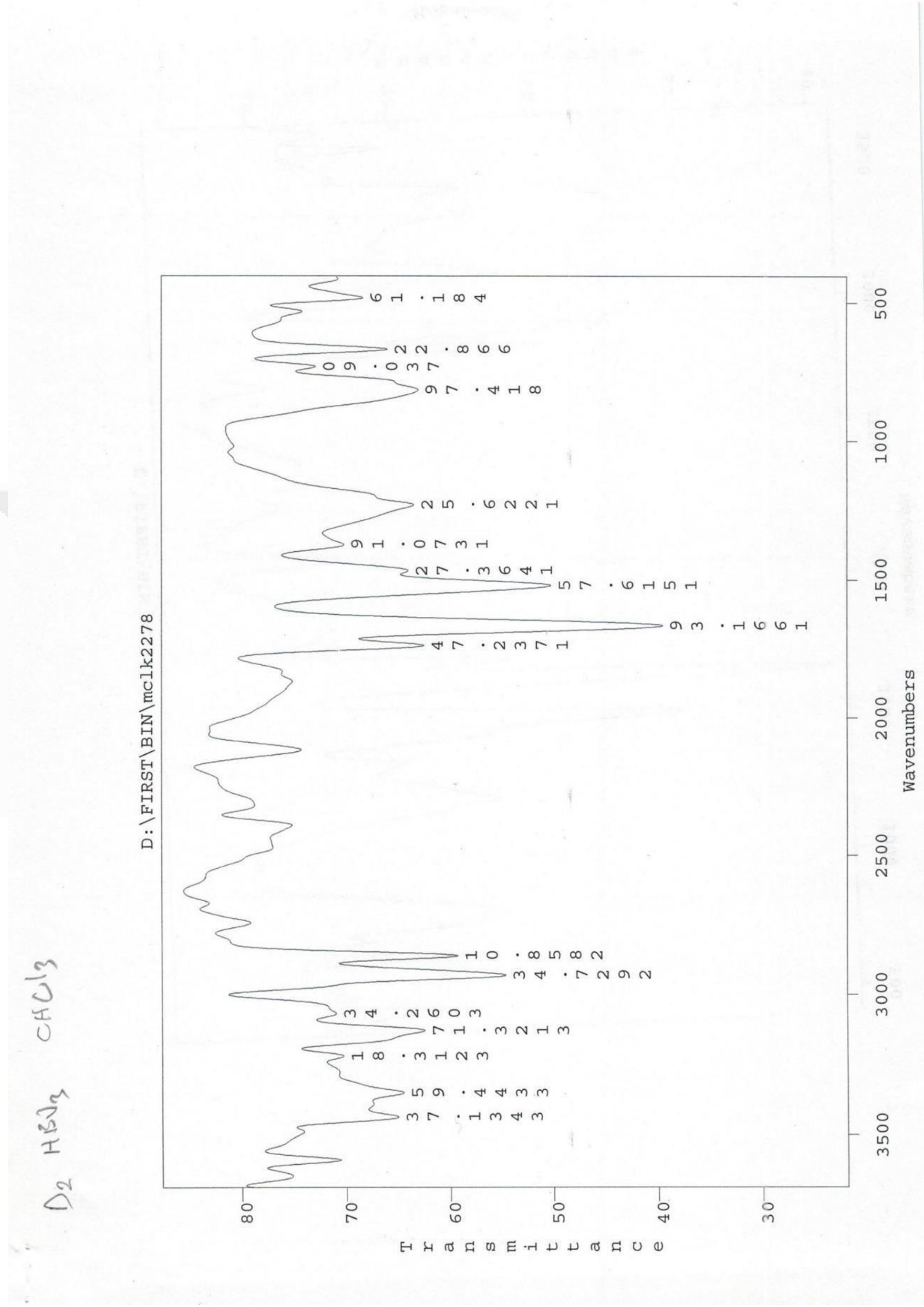
D:\FIRST\BIN\mclk2278
Wavenumbers
Transmittance

-

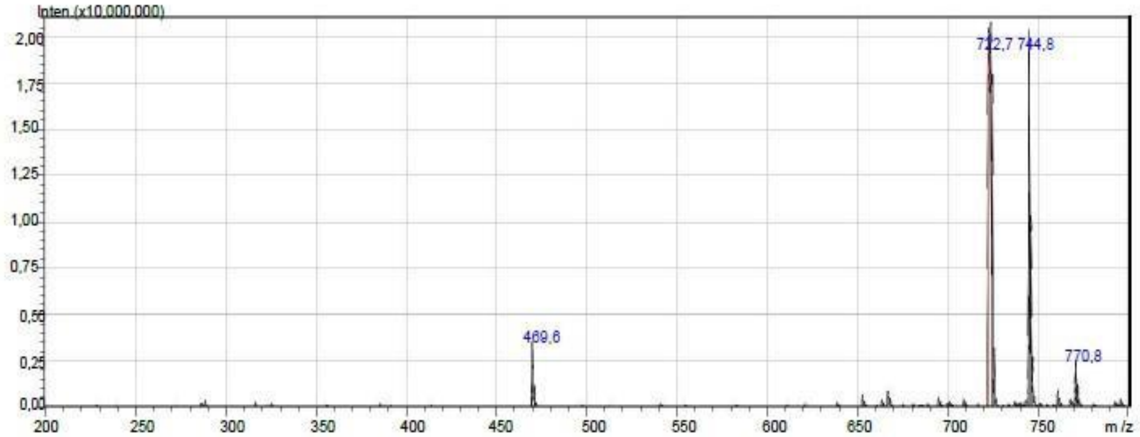
Inten.(x10,000,000)
722,7
744,8
469,6
770,8
m/z

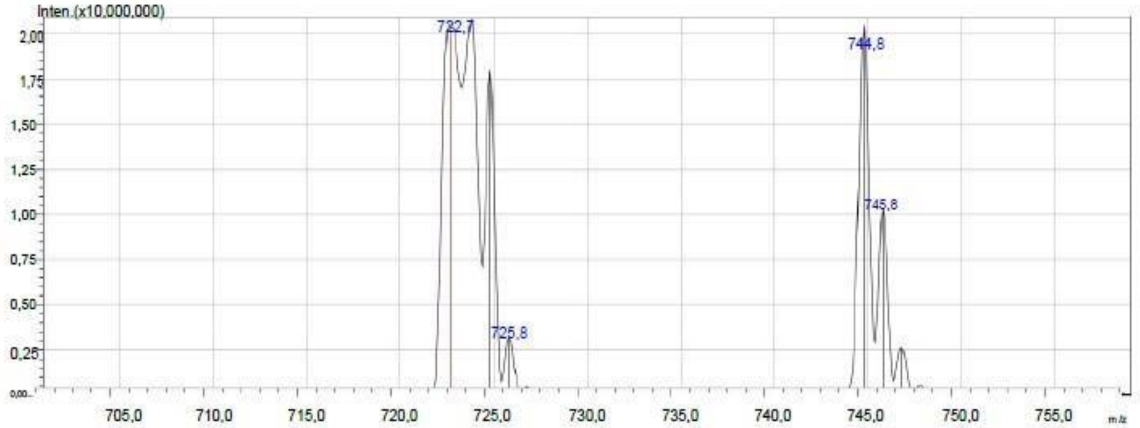
Inten.(x10,000,000)
722,7
744,8
745,8
725,8
m/z

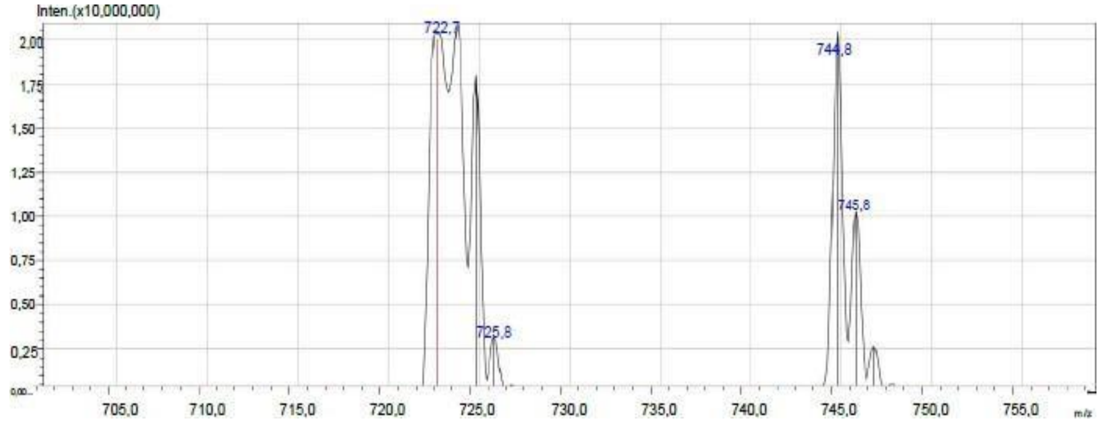
Inten.(x10,000,000)
722,7
744,8
745,8
725,8
m/z

D2E JELATÖRÜNÜN NMR, IR VE MS SPEKTRUMLARI

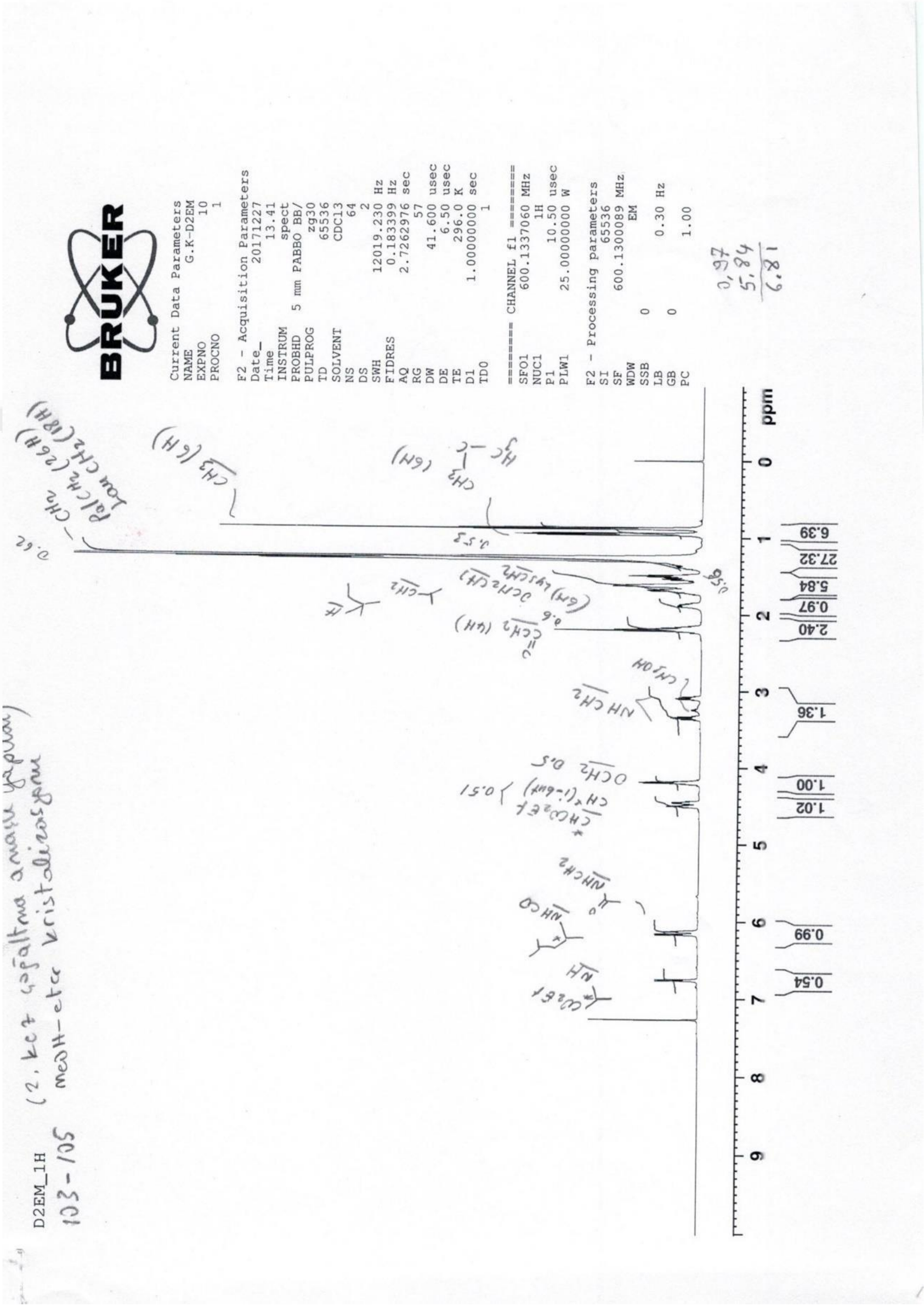

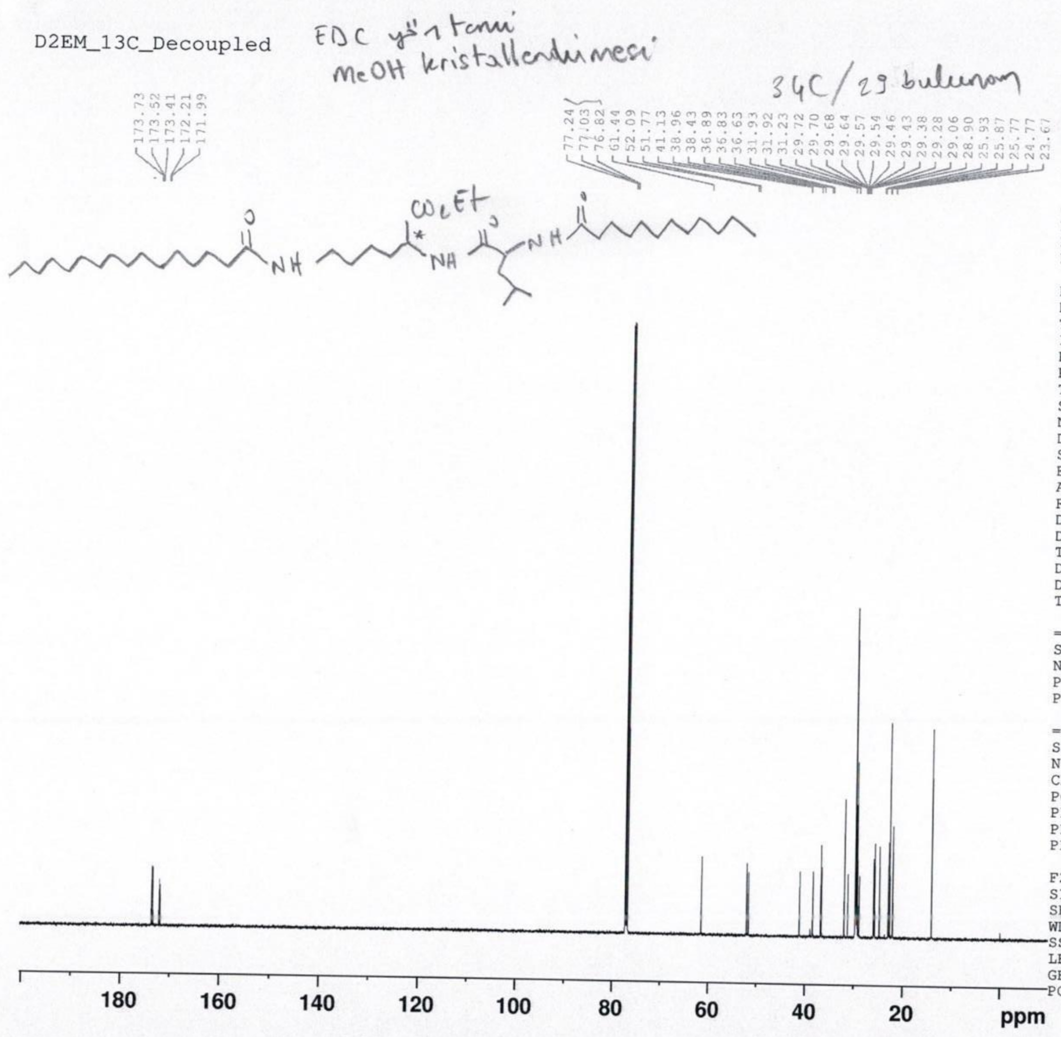

BRUKER

Current Data Parameters
NAME G.K-D2EM
EXPNO 11
PROCNO 1

F2 - Acquisition Parameters
Date_ 20171229
Time 11.03
INSTRUM spect
PROBHD 5 mm PABBO BB/
PULPROG zgpg30
TD 65536
SOLVENT CDCl3
NS 2048
DS 4
SWH 36057.691 Hz
FIDRES 0.550197 Hz
AQ 0.9087659 sec
RG 2050
DW 13.867 usec
DE 6.50 usec
TE 296.0 K
D1 2.00000000 sec
D11 0.03000000 sec
TD0 1

======== CHANNEL f1 ========
SFO1 150.9178981 MHz
NUC1 13C
P1 10.50 usec
PLW1 100.00000000 W

======== CHANNEL f2 ========
SFO2 600.1324005 MHz
NUC2 1H
CPDPRG[2 waltz16
PCPD2 70.00 usec
PLW2 25.00000000 W
PLW12 0.56250000 W
PLW13 0.28292999 W

F2 - Processing parameters
SI 32768
SF 150.9028085 MHz
WDW EM
SSB 0
LB 1.00 Hz
GB 0
PC 1.40

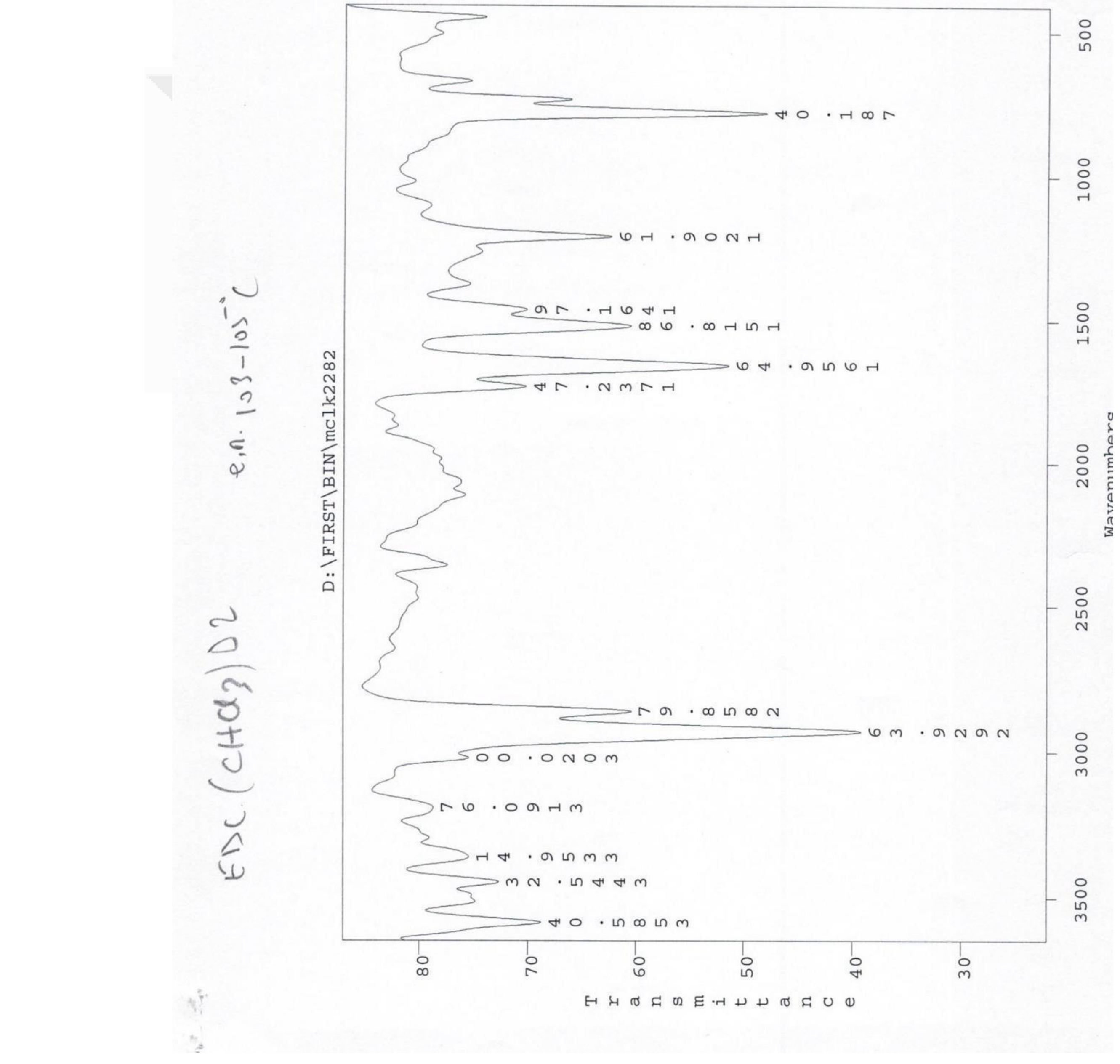
EDC (CHCl3) D2
e.n. 103-105°C
D:\FIRST\BIN\mclk2282
Transmittance
Wavenumbers
3500
3000
2500
2000
1500
1000
500
80
70
60
50
40
30

D3 JELATÖRÜNÜN NMR, IR VE MS SPEKTRUMLARI

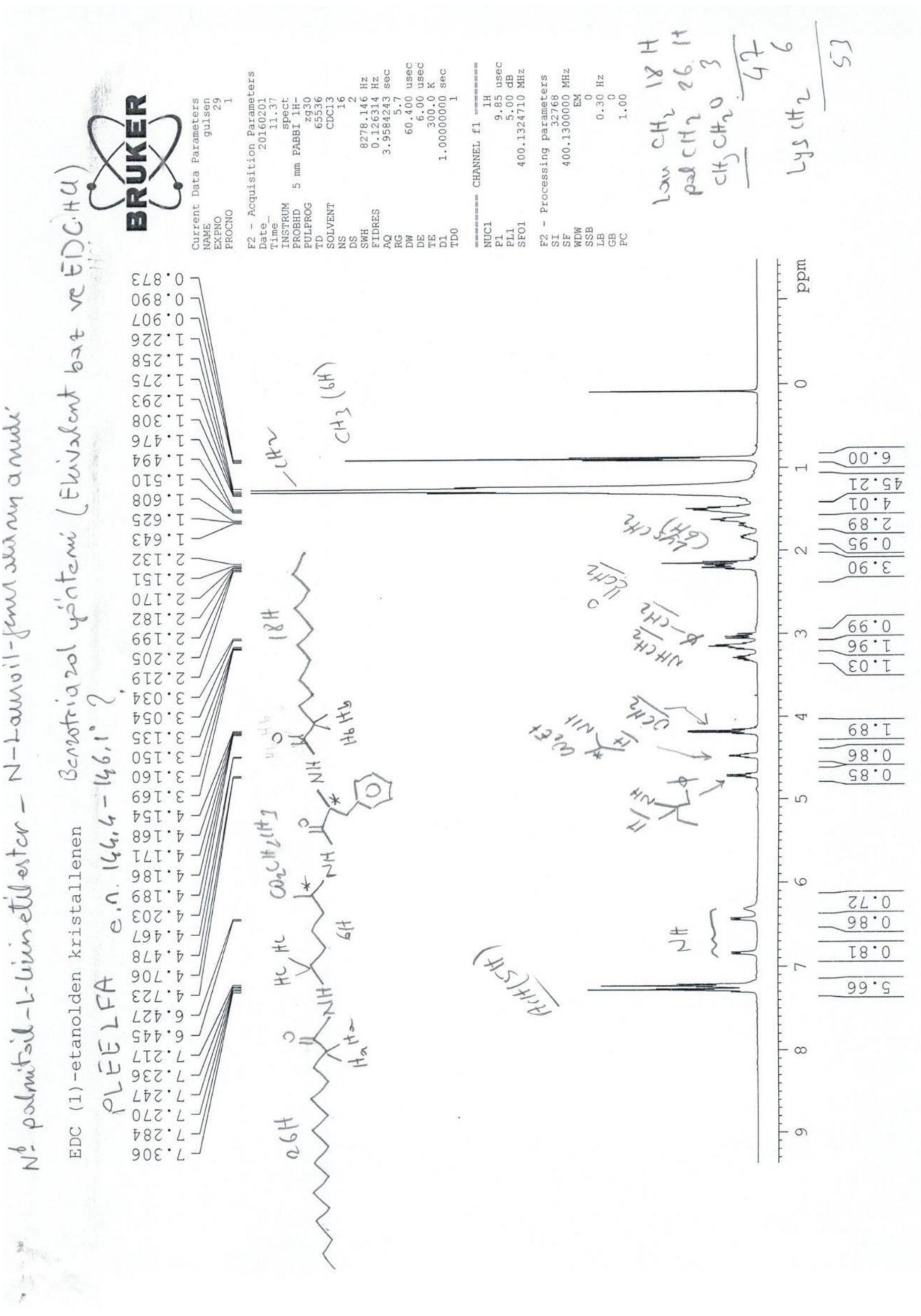

PLEELFA

EDC (3)-etanolden kristallenen

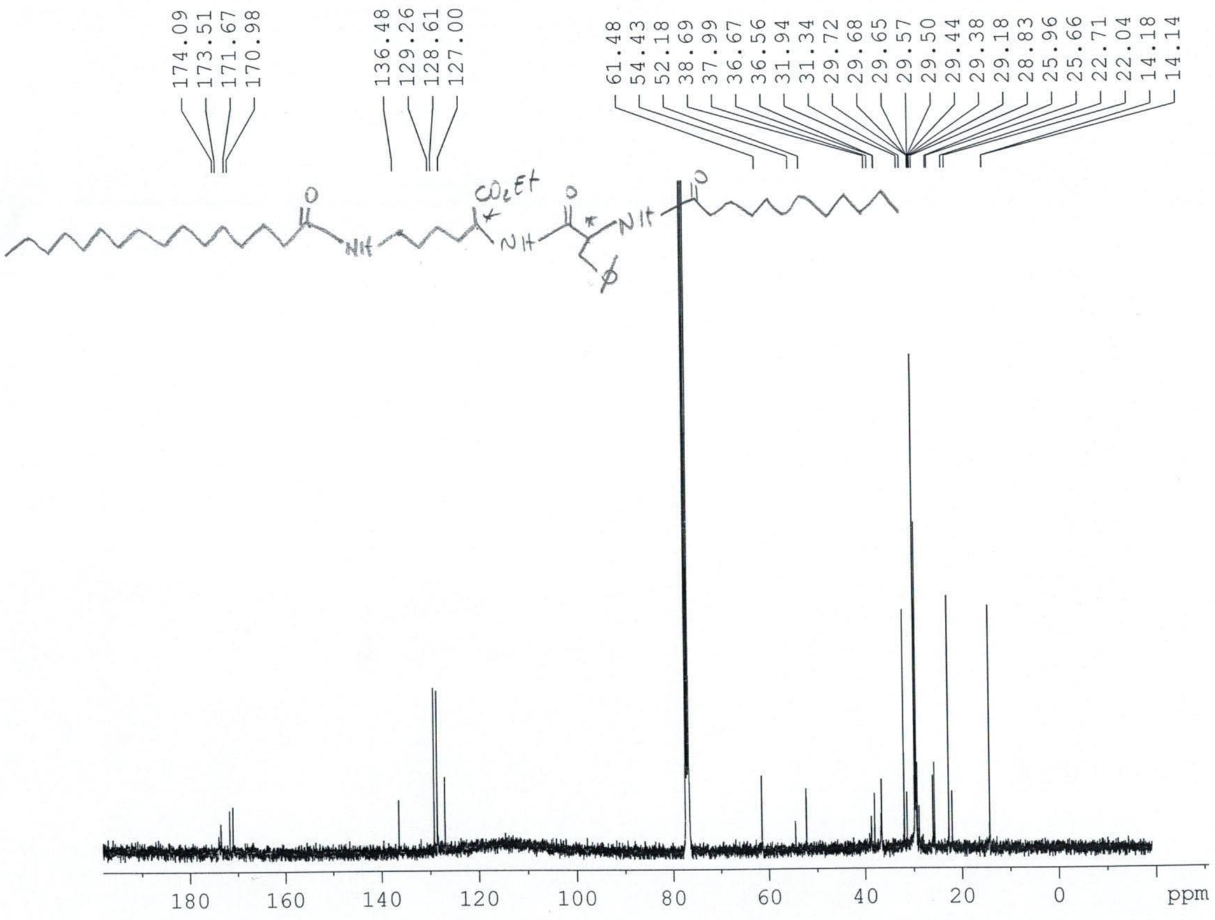

BRUKER

Current Data Parameters

NAME	gulsen
EXPNO	32
PROCNO	1

F2 - Acquisition Parameters

Date_	20160201
Time	23.59
INSTRUM	spect
PROBHD	5 mm PABBI 1H-
PULPROG	zgpg30
TD	65536
SOLVENT	CDCl3
NS	10240
DS	4
SWH	23980.814 Hz
FIDRES	0.365918 Hz
AQ	1.3664756 sec
RG	1024
DW	20.850 usec
DE	6.00 usec
TE	300.0 K
D1	2.00000000 sec
d11	0.03000000 sec
DELTA	1.89999998 sec
TD0	1

======== CHANNEL f1 ========

NUC1	13C
P1	13.33 usec
PL1	-6.00 dB
SFO1	100.6228298 MHz

======== CHANNEL f2 ========

CPDPRG2	waltz16
NUC2	1H
PCPD2	70.00 usec
PL2	5.00 dB
PL12	22.03 dB
PL13	27.00 dB
SFO2	400.1316005 MHz

F2 - Processing parameters

SI	32768
SF	100.6127690 MHz
WDW	EM
SSB	0
LB	1.00 Hz
GB	0
PC	1.40

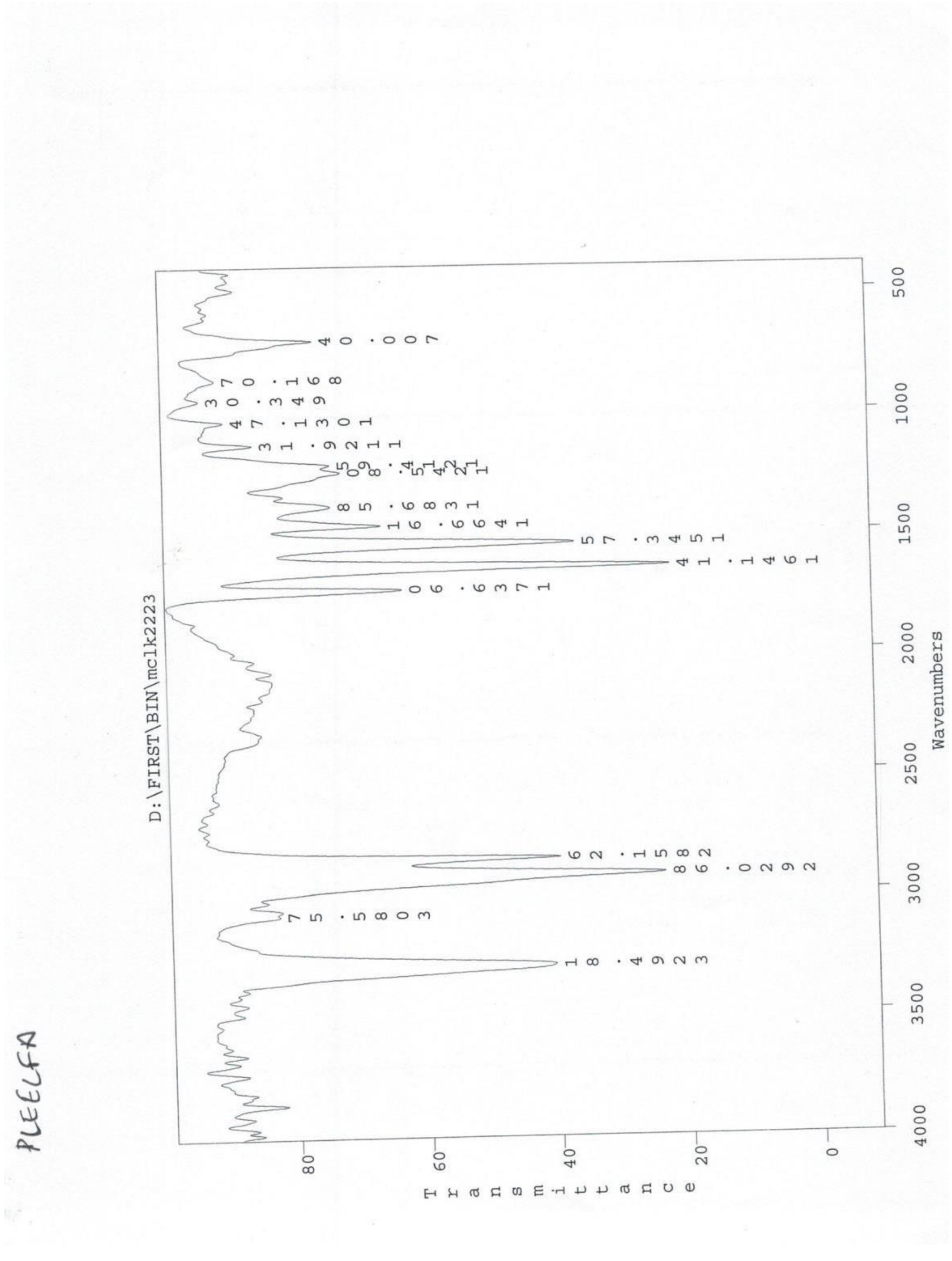
PLEELFA
D:\FIRST\BIN\mclk2223
Transmittance
80
60
40
20
0
4000
3500
3000
2500
2000
1500
1000
500
Wavenumbers
18.4923
75.5803
86.0292
62.1582
06.6371
41.1461
57.3451
16.6641
85.6831
08.5421
59.4121
31.9211
47.1301
30.349
70.168
40.007

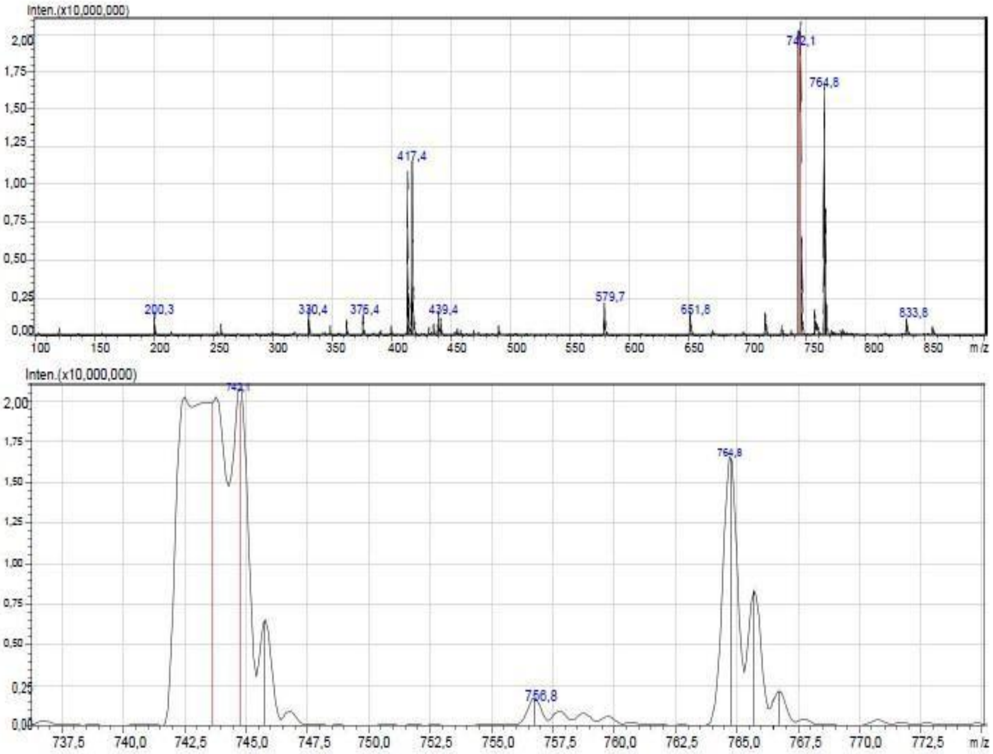
Inten.(x10,000,000)
742,1
764,8
417,4
200,3
330,4
376,4
439,4
579,7
651,8
833,8
m/z
Inten.(x10,000,000)
756,8
764,8
m/z

D4 JELATÖRÜNÜN NMR, IR VE MS SPEKTRUMLARI

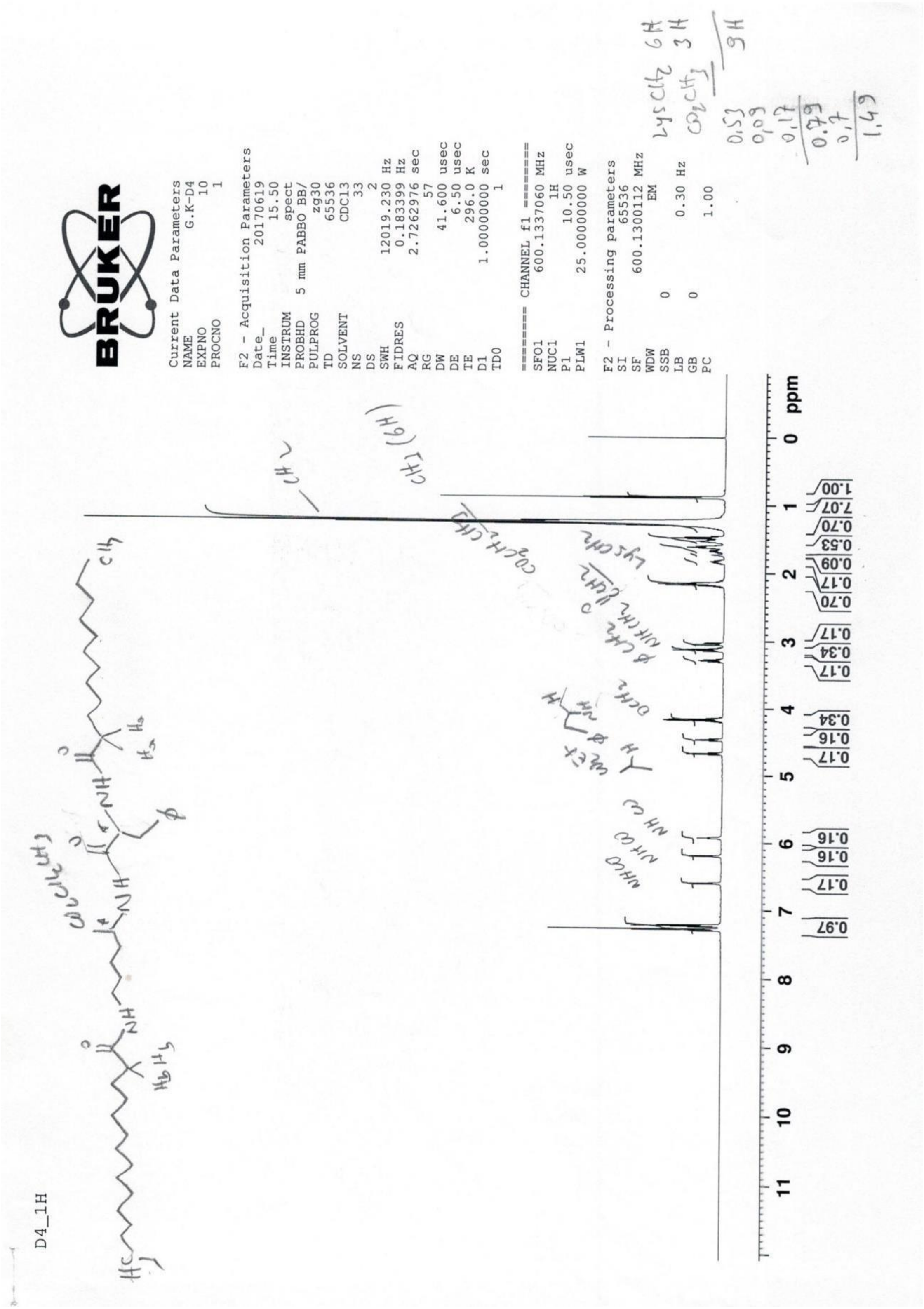

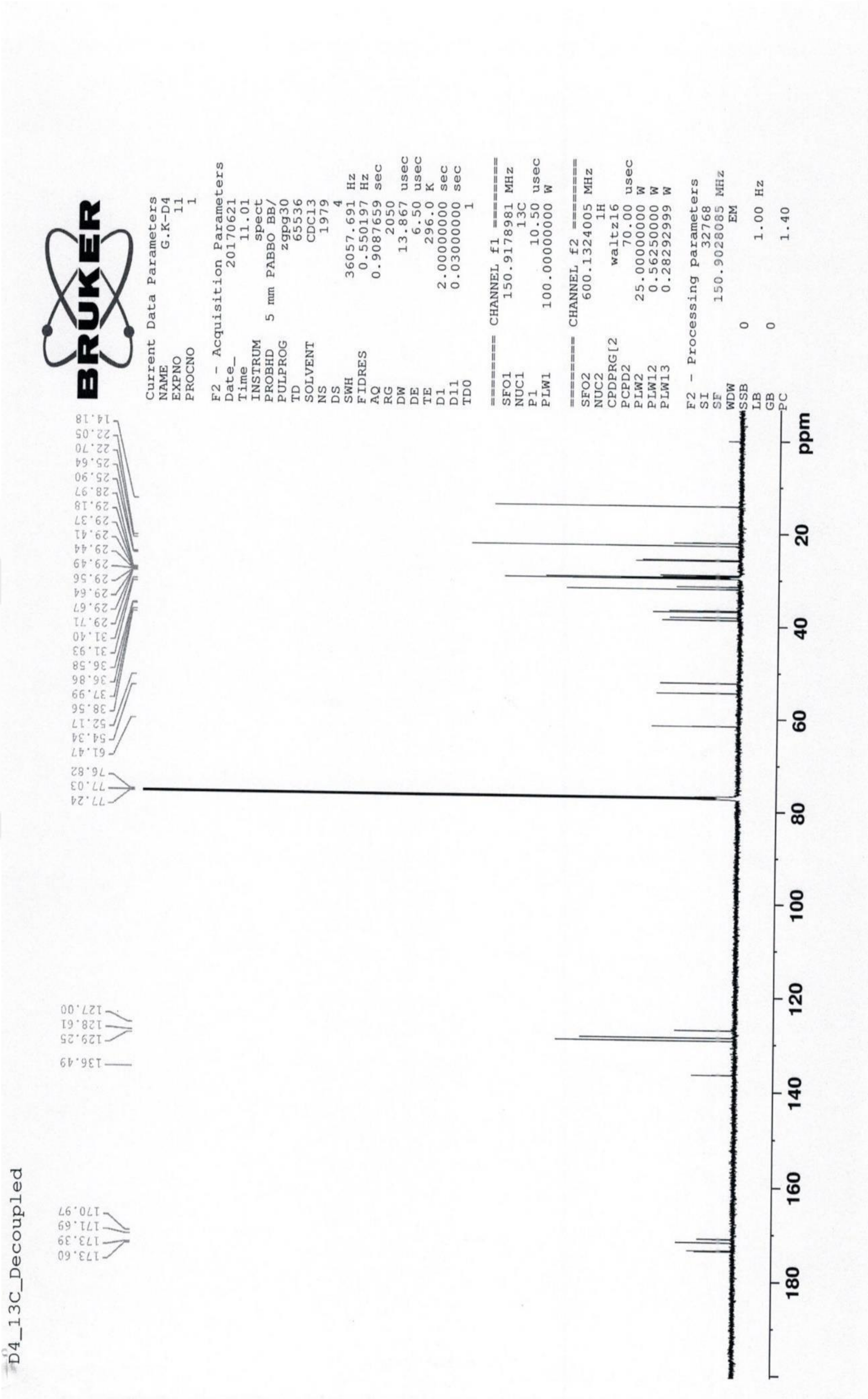
D4_13C_Decoupled
14.18
22.05
22.70
25.64
25.90
28.97
29.18
29.37
29.41
29.44
29.49
29.56
29.64
29.67
29.71
31.40
31.93
36.58
36.86
37.99
38.56
52.17
54.34
61.47
76.82
77.03
77.24
127.00
128.61
129.25
136.49
170.97
171.69
173.36
173.60
180
160
140
120
100
80
60
40
20
ppm
BRUKER
Current Data Parameters
NAME G.K-D4
EXPNO 11
PROCNO 1
F2 - Acquisition Parameters
Date_ 20170621
Time 11.01
INSTRUM spect
PROBHD 5 mm PABBO BB/
PULPROG zgpg30
TD 65536
SOLVENT CDCl3
NS 1979
DS 4
SWH 36057.691 Hz
FIDRES 0.550197 Hz
AQ 0.9087659 sec
RG 2050
DW 13.867 usec
DE 6.50 usec
TE 296.0 K
D1 2.00000000 sec
D11 0.03000000 sec
TD0 1
======== CHANNEL f1 ========
SFO1 150.9178981 MHz
NUC1 13C
P1 10.50 usec
PLW1 100.00000000 W
======== CHANNEL f2 ========
SFO2 600.1324005 MHz
NUC2 1H
CPDPRG[2 waltz16
PCPD2 70.00 usec
PLW2 25.00000000 W
PLW12 0.56250000 W
PLW13 0.28292999 W
F2 - Processing parameters
SI 32768
SF 150.9028085 MHz
WDW EM
SSB 0
LB 1.00 Hz
GB 0
PC 1.40

-

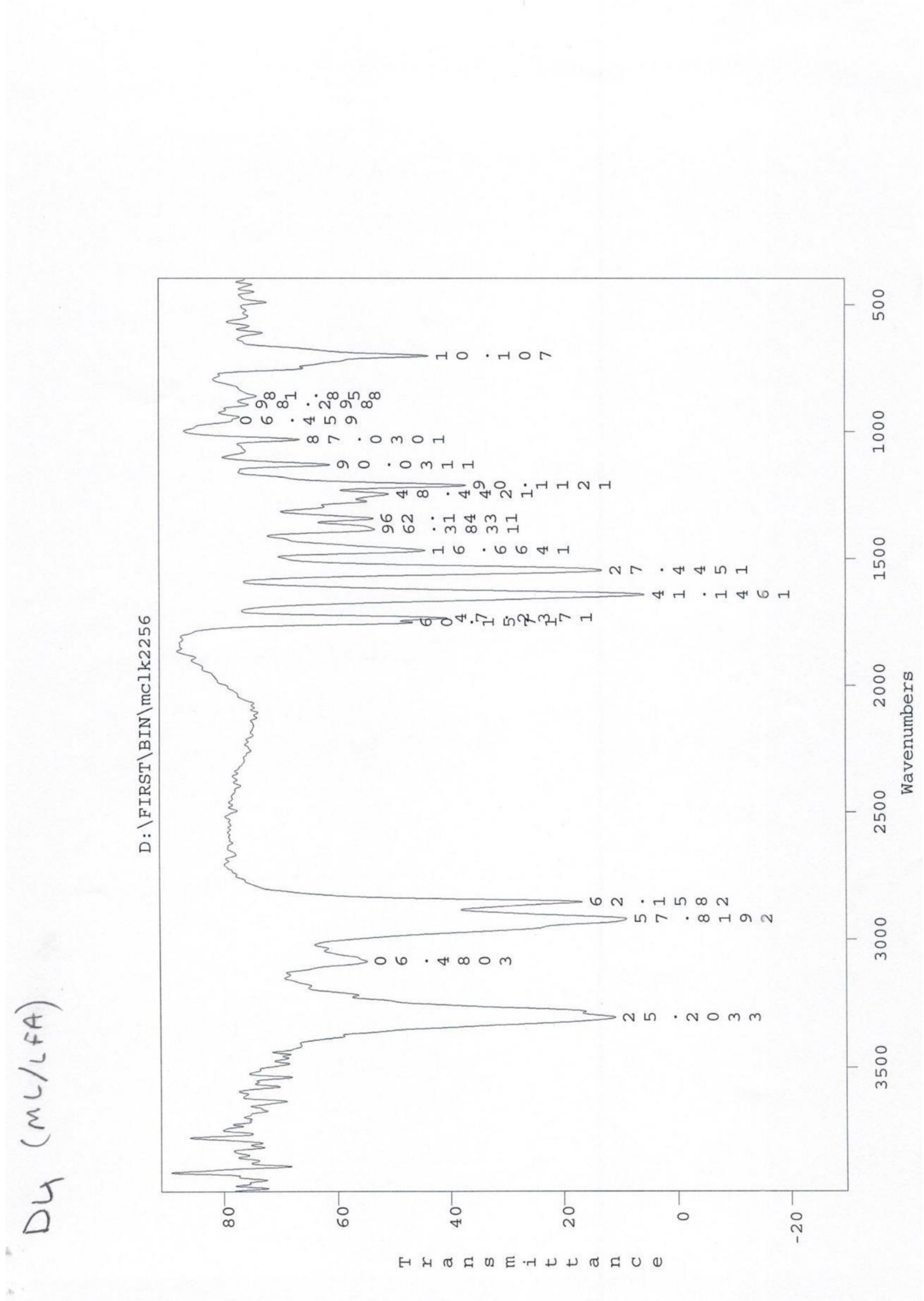
D4 (ML/LFA)
D:\FIRST\BIN\mclk2256
Transmittance
Wavenumbers

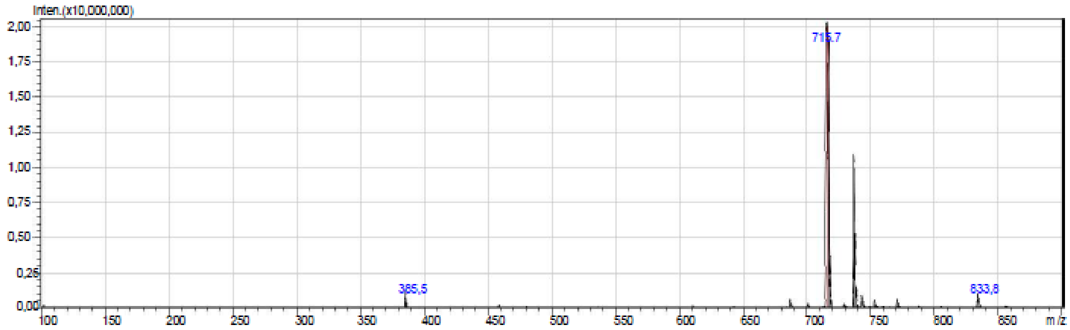
Inten.(x10,000,000)
2,00
1,75
1,50
1,25
1,00
0,75
0,50
0,25
0,00
715,7
385,5
833,8
100
150
200
250
300
350
400
450
500
550
600
650
700
750
800
850
m/z

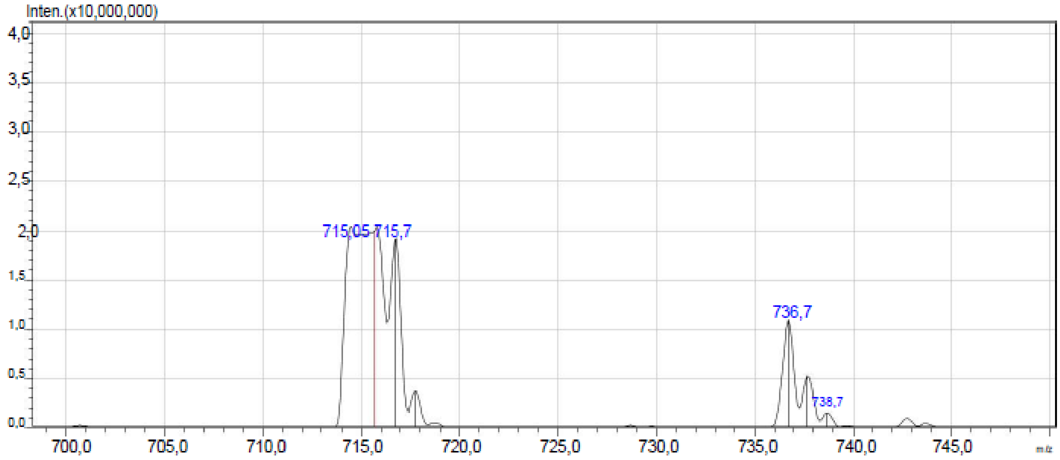
Inten.(x10,000,000)
4,0
3,5
3,0
2,5
2,0
1,5
1,0
0,5
0,0
715,05
715,7
736,7
738,7
700,0
705,0
710,0
715,0
720,0
725,0
730,0
735,0
740,0
745,0

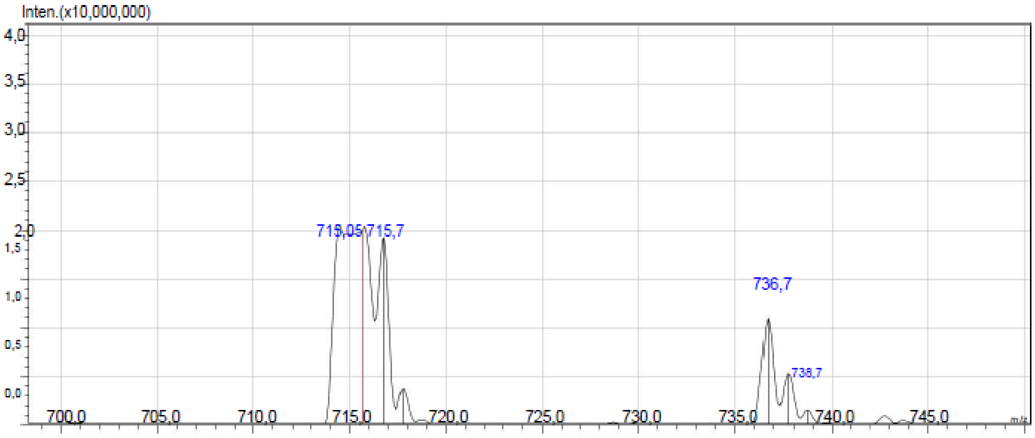
Inten.(x10,000,000)
4,0
3,5
3,0
2,5
2,0
1,5
1,0
0,5
0,0
715,05
715,7
736,7
738,7
700,0
705,0
710,0
715,0
720,0
725,0
730,0
735,0
740,0
745,0

-

D5 JELATÖRÜNÜN NMR, IR VE MS SPEKTRUMLARI

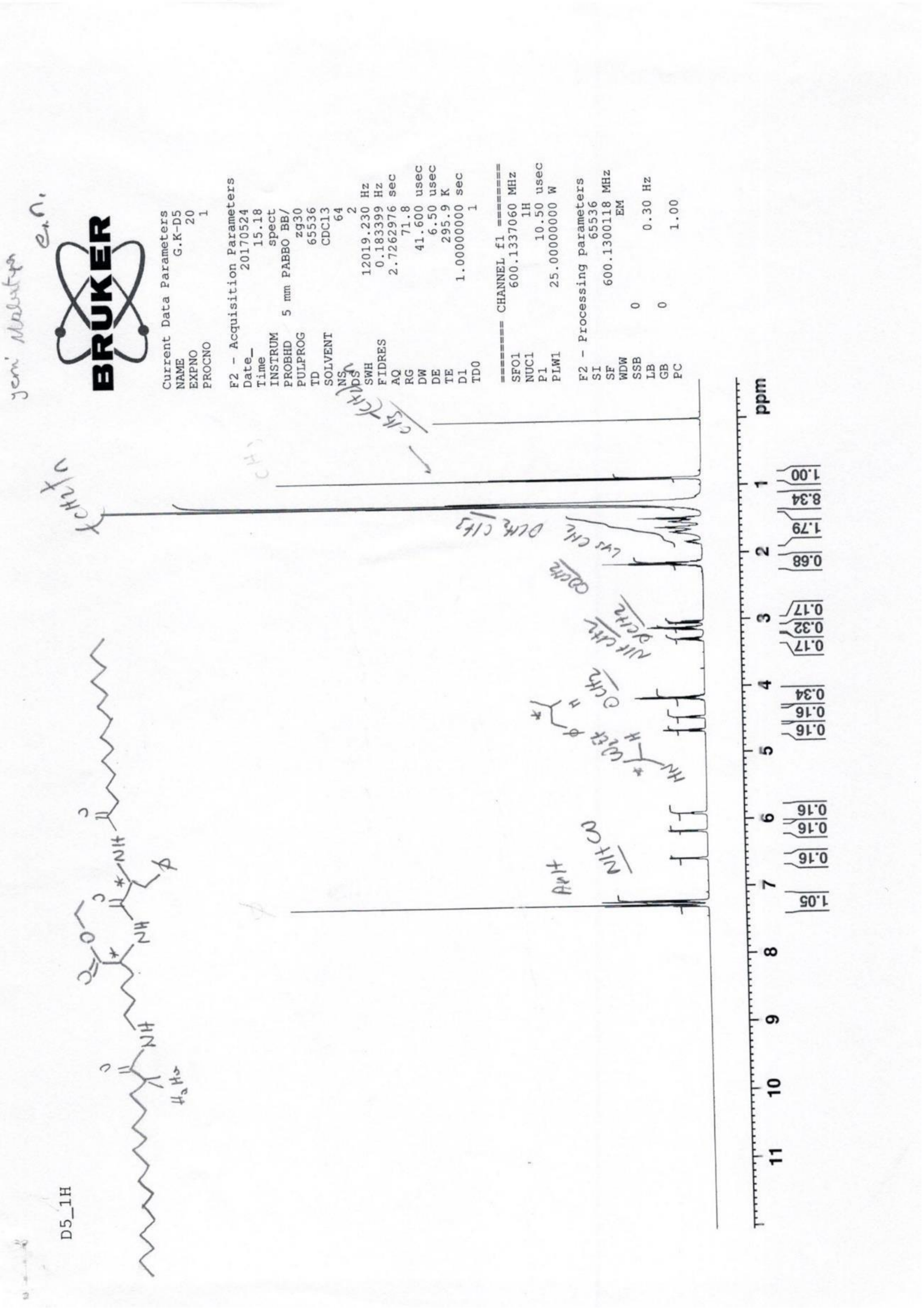

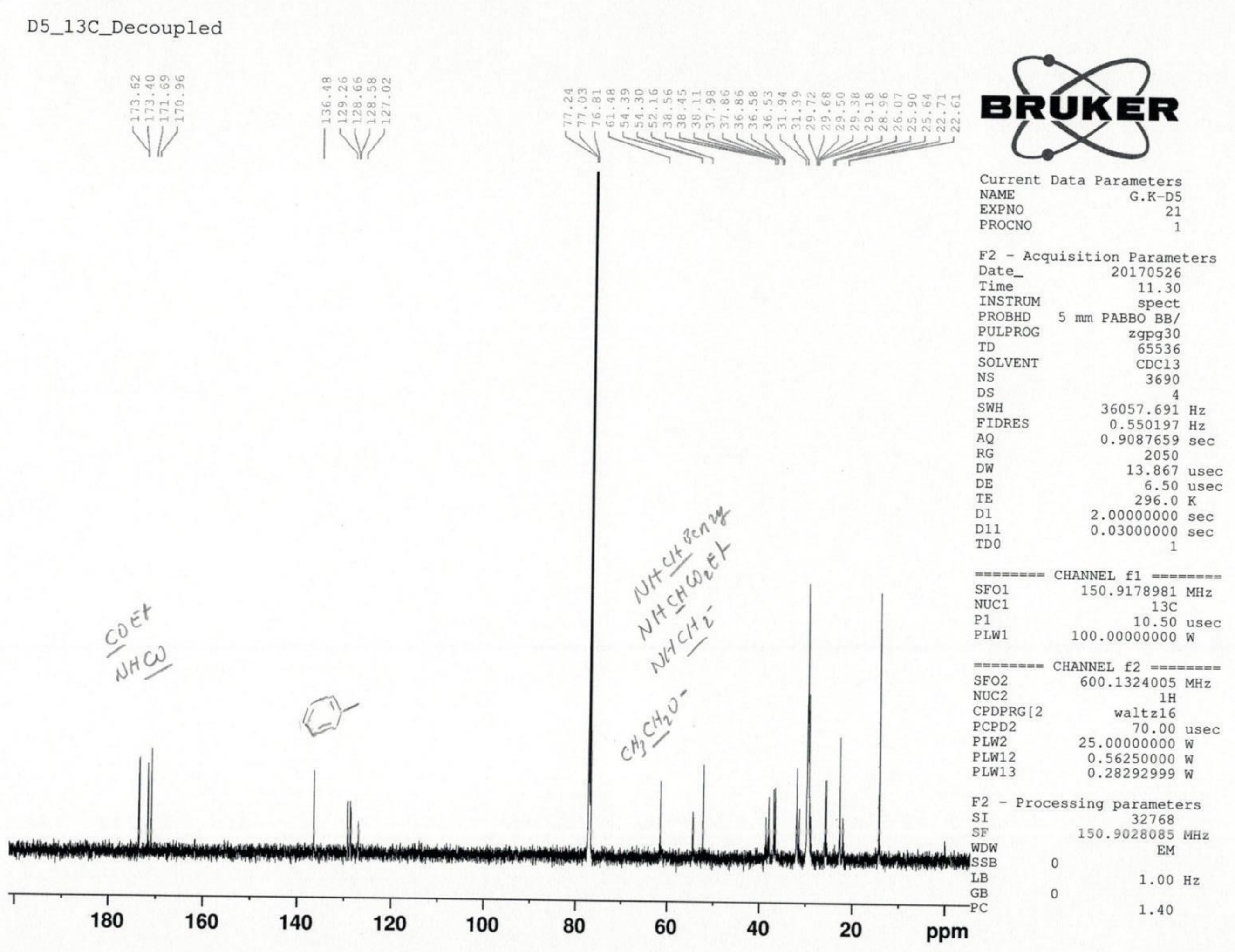
D5_13C_Decoupled
173.62
173.40
171.69
170.96
136.48
129.26
128.66
128.58
127.02
77.24
77.03
76.81
61.48
54.39
54.30
52.16
38.56
38.45
38.11
37.98
37.86
36.86
36.58
36.53
31.94
31.39
29.72
29.68
29.50
29.38
29.18
28.96
26.07
25.90
25.64
22.71
22.61
BRUKER
Current Data Parameters
NAME G.K-D5
EXPNO 21
PROCNO 1
F2 - Acquisition Parameters
Date_ 20170526
Time 11.30
INSTRUM spect
PROBHD 5 mm PABBO BB/
PULPROG zgpg30
TD 65536
SOLVENT CDCl3
NS 3690
DS 4
SWH 36057.691 Hz
FIDRES 0.550197 Hz
AQ 0.9087659 sec
RG 2050
DW 13.867 usec
DE 6.50 usec
TE 296.0 K
D1 2.00000000 sec
D11 0.03000000 sec
TD0 1
======== CHANNEL f1 ========
SFO1 150.9178981 MHz
NUC1 13C
P1 10.50 usec
PLW1 100.00000000 W
======== CHANNEL f2 ========
SFO2 600.1324005 MHz
NUC2 1H
CPDPRG[2 waltz16
PCPD2 70.00 usec
PLW2 25.00000000 W
PLW12 0.56250000 W
PLW13 0.28292999 W
F2 - Processing parameters
SI 32768
SF 150.9028085 MHz
WDW EM
SSB 0
LB 1.00 Hz
GB 0
PC 1.40
180
160
140
120
100
80
60
40
20
ppm

-

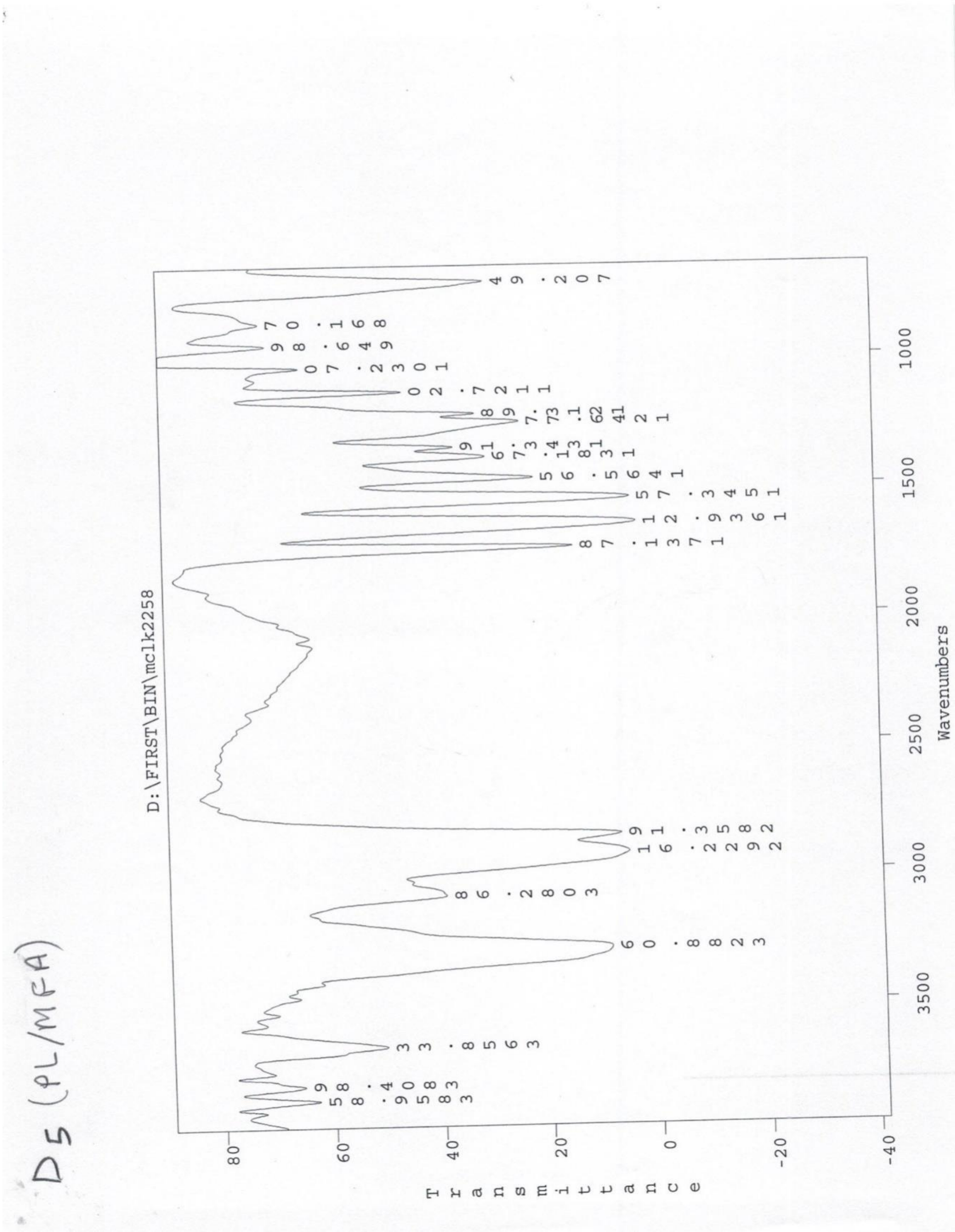
D5 (PL/MFA)
D:\FIRST\BIN\mclk2258
Transmittance
Wavenumbers

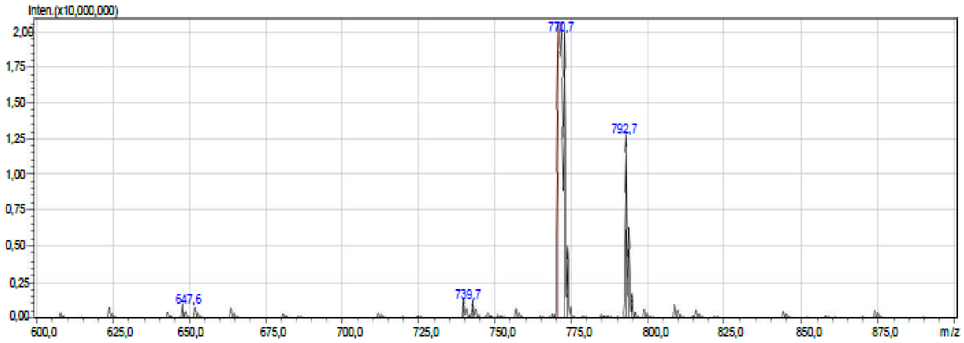

Inten.(x10,000,000)
770,7
792,7
647,6
739,7
m/z

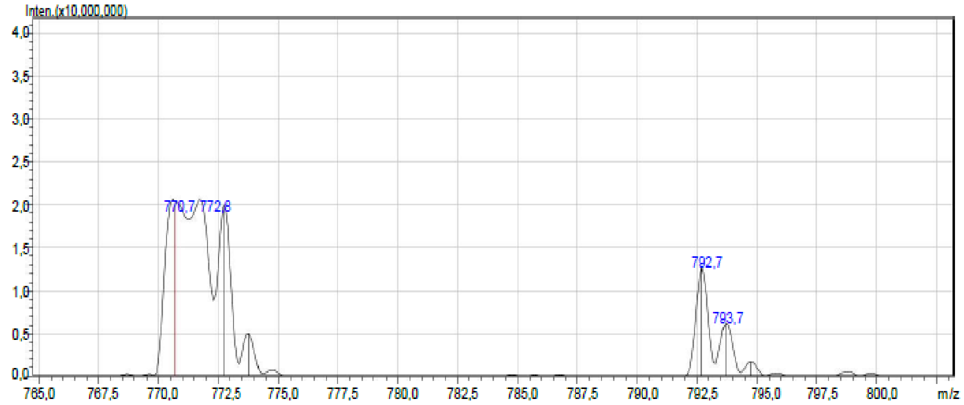

Inten.(x10,000,000)
770,7/772,8
792,7
793,7
m/z

-

D6 JELATÖRÜNÜN NMR, IR VE MS SPEKTRUMLARI

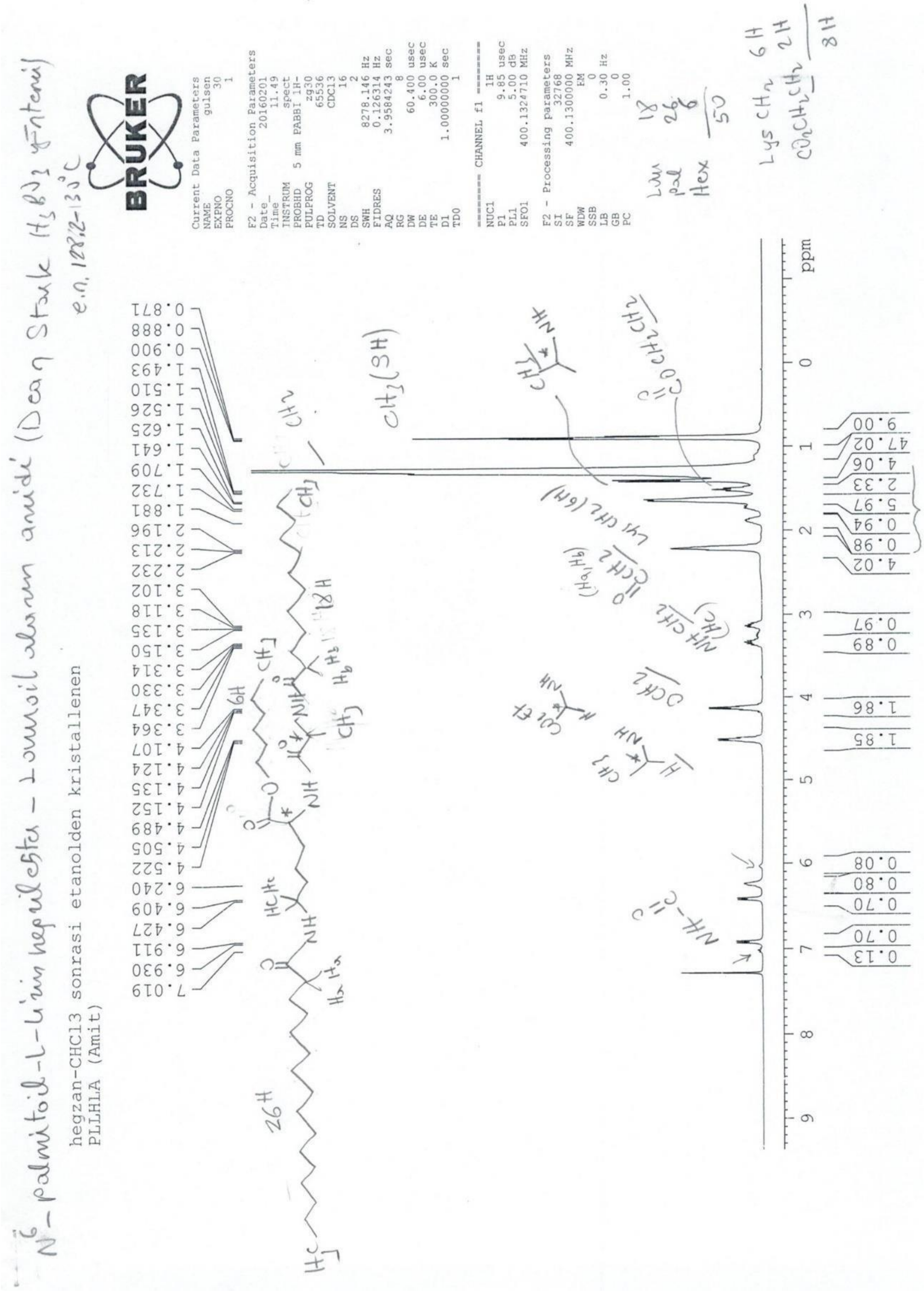

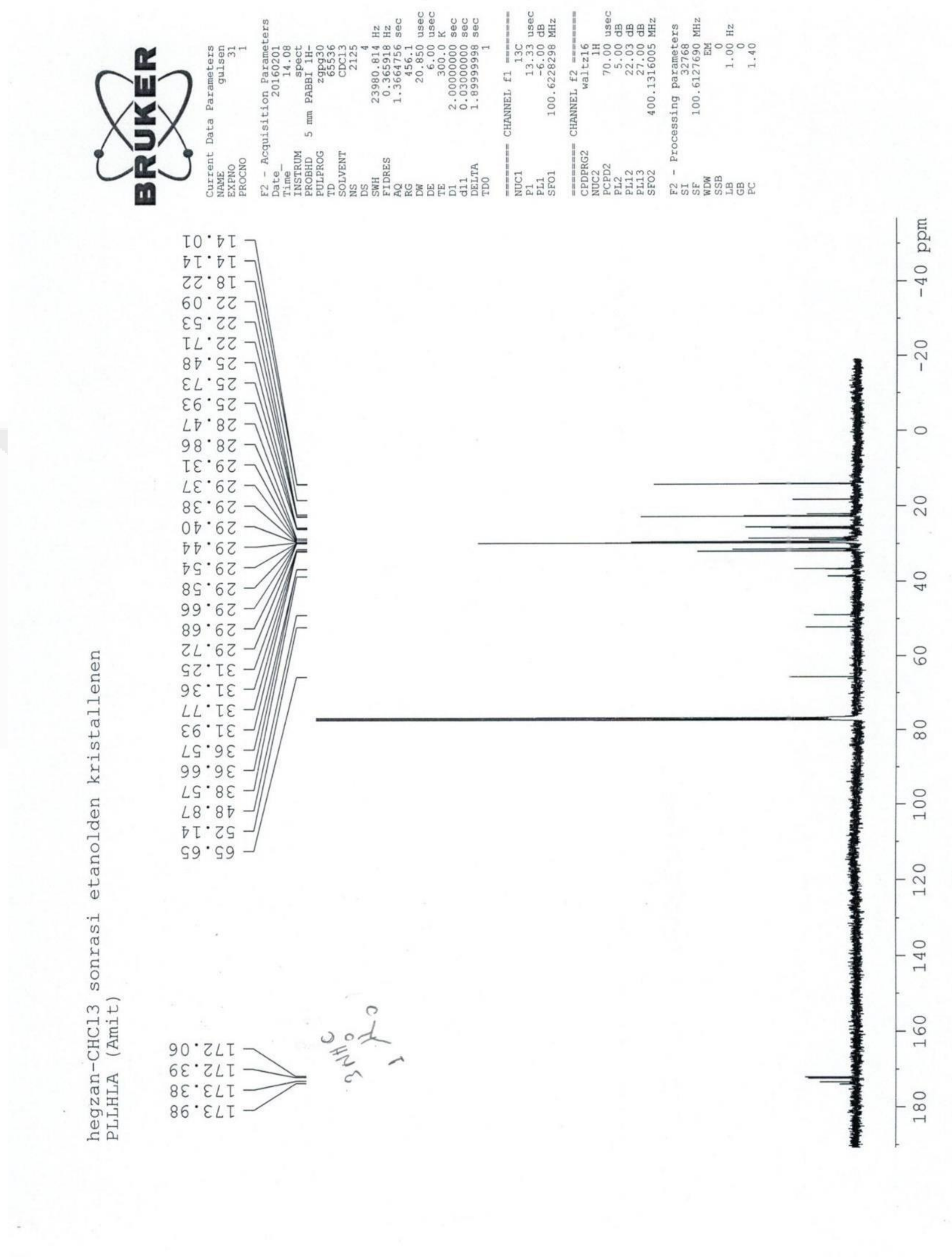
hegzan-CHCl3 sonrasi etanolden kristallenen
PLLHLA (Amit)
173.98
173.38
172.39
172.06
65.65
52.14
48.87
38.57
36.66
36.57
31.93
31.77
31.36
31.25
29.72
29.68
29.66
29.58
29.54
29.44
29.40
29.38
29.37
29.31
28.86
28.47
25.93
25.73
25.48
22.71
22.53
22.09
18.22
14.14
14.01
BRUKER
Current Data Parameters
NAME gulsen
EXPNO 31
PROCNO 1
F2 - Acquisition Parameters
Date_ 20160201
Time 14.08
INSTRUM spect
PROBHD 5 mm PABBI 1H-
PULPROG zgpg30
TD 65536
SOLVENT CDCl3
NS 2125
DS 4
SWH 23980.814 Hz
FIDRES 0.365918 Hz
AQ 1.3664756 sec
RG 456.1
DW 20.850 usec
DE 6.00 usec
TE 300.0 K
D1 2.00000000 sec
d11 0.03000000 sec
DELTA 1.89999998 sec
TD0 1
======== CHANNEL f1 ========
NUC1 13C
P1 13.33 usec
PL1 -6.00 dB
SFO1 100.6228298 MHz
======== CHANNEL f2 ========
CPDPRG2 waltz16
NUC2 1H
PCPD2 70.00 usec
PL2 5.00 dB
PL12 22.03 dB
PL13 27.00 dB
SFO2 400.1316005 MHz
F2 - Processing parameters
SI 32768
SF 100.6127690 MHz
WDW EM
SSB 0
LB 1.00 Hz
GB 0
PC 1.40
180
160
140
120
100
80
60
40
20
0
-20
-40 ppm

-

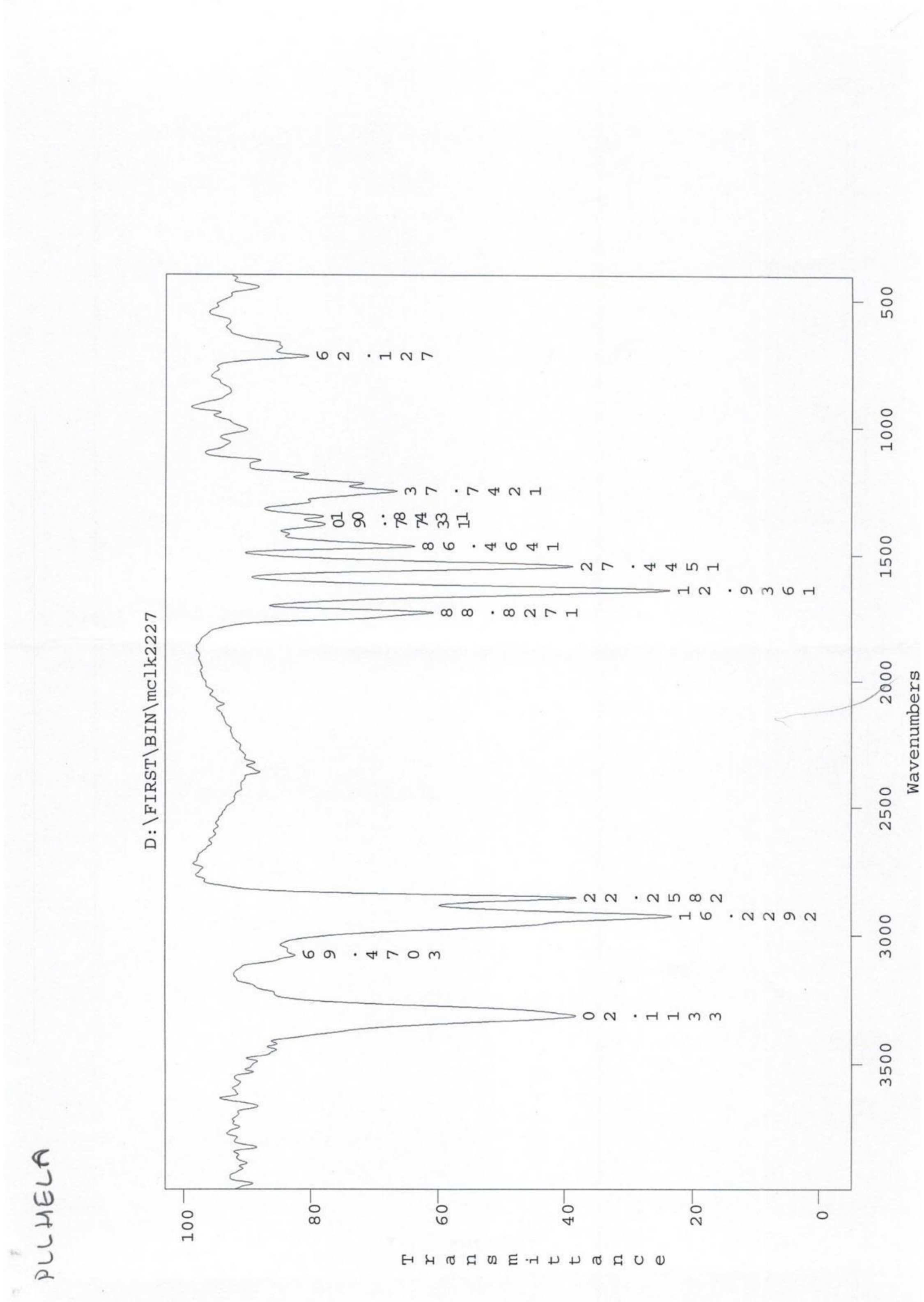
PULHELA
D:\FIRST\BIN\mclk2227
Transmittance
Wavenumbers
3311.20
3074.96
2922.61
2852.22
1728.88
1639.21
1544.72
1464.68
1247.73
721.26
100
80
60
40
20
0
3500
3000
2500
2000
1500
1000
500

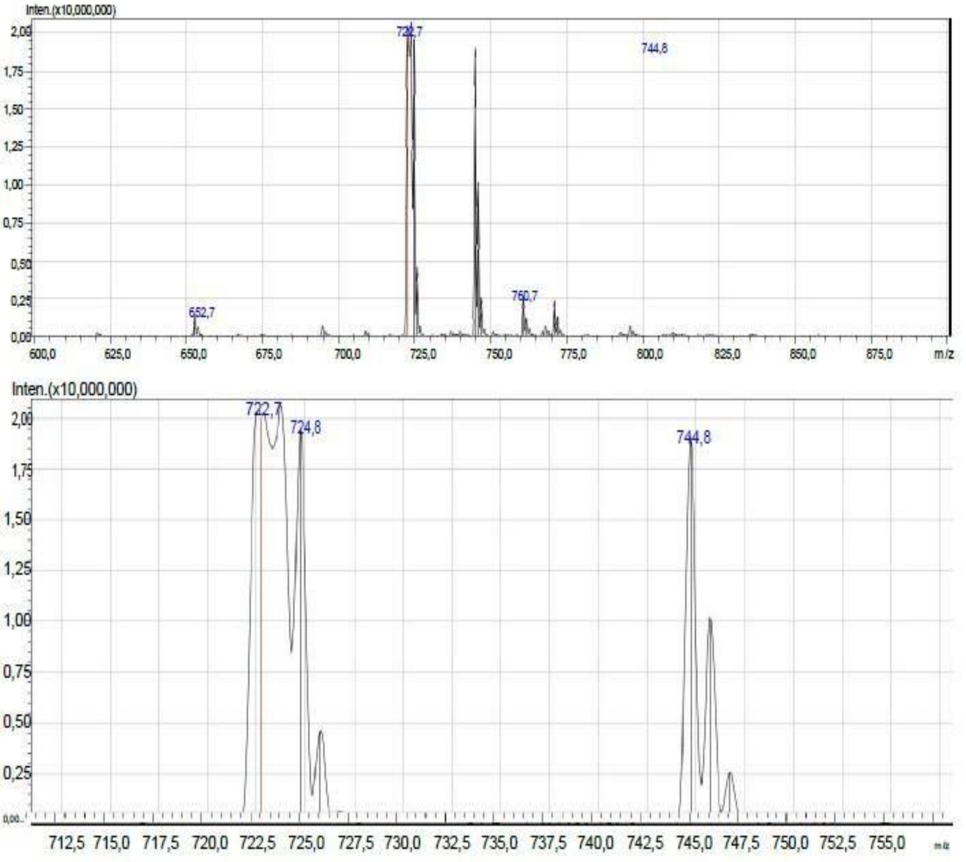
Inten.(x10,000,000)
722,7
744,8
760,7
652,7
m/z
Inten.(x10,000,000)
722,7
724,8
744,8
m/z

-

D7 JELATÖRÜNÜN NMR, IR VE MS SPEKTRUMLARI

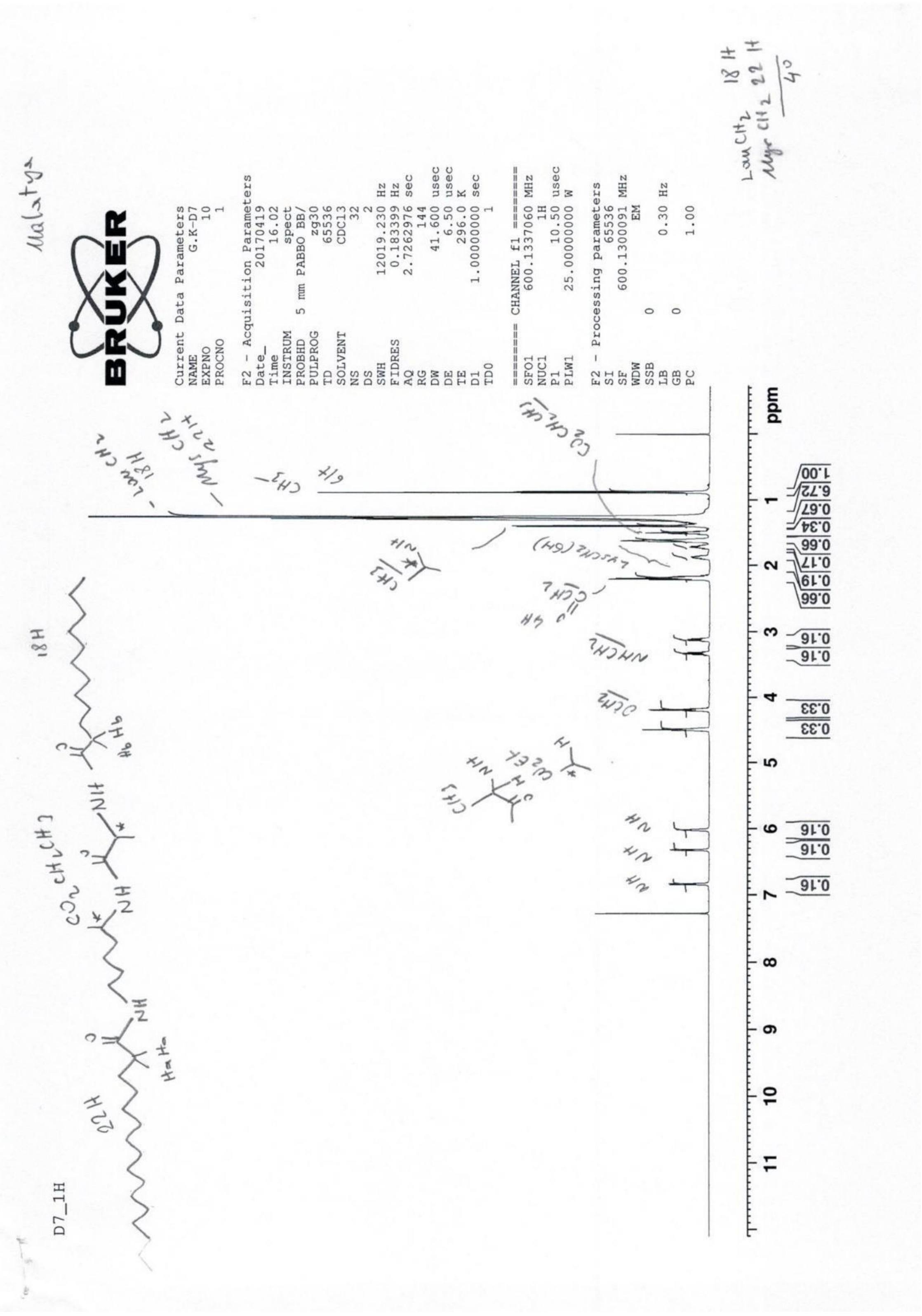

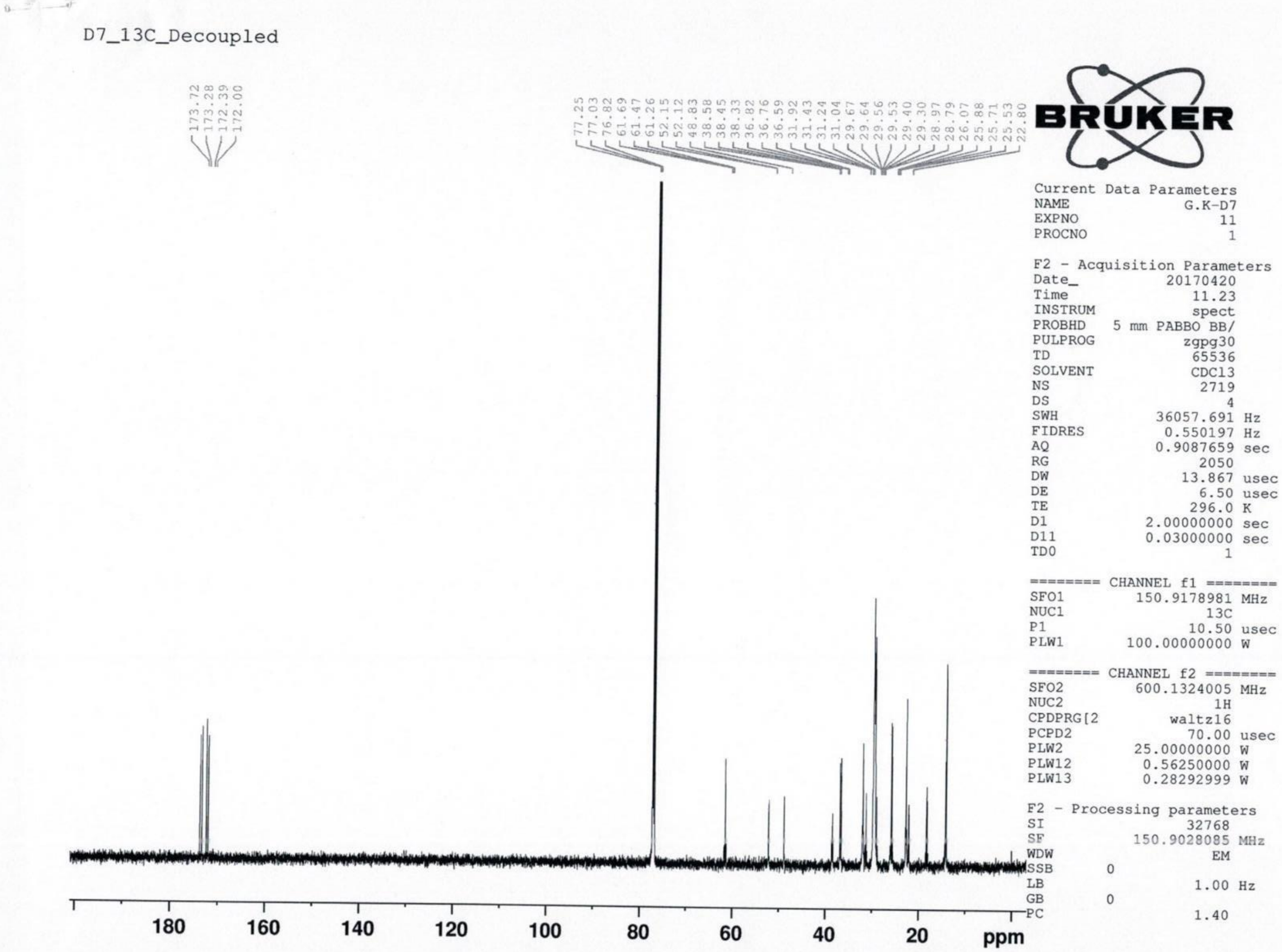
D7_13C_Decoupled
173.72
173.28
172.39
172.00
77.25
77.03
76.82
61.69
61.47
61.26
52.15
52.12
48.83
38.58
38.45
38.33
36.82
36.76
36.59
31.92
31.43
31.24
31.04
29.67
29.64
29.56
29.53
29.40
29.30
28.97
28.79
26.07
25.88
25.71
25.53
22.80
BRUKER
Current Data Parameters
NAME G.K-D7
EXPNO 11
PROCNO 1
F2 - Acquisition Parameters
Date_ 20170420
Time 11.23
INSTRUM spect
PROBHD 5 mm PABBO BB/
PULPROG zgpg30
TD 65536
SOLVENT CDCl3
NS 2719
DS 4
SWH 36057.691 Hz
FIDRES 0.550197 Hz
AQ 0.9087659 sec
RG 2050
DW 13.867 usec
DE 6.50 usec
TE 296.0 K
D1 2.00000000 sec
D11 0.03000000 sec
TD0 1
======== CHANNEL f1 ========
SFO1 150.9178981 MHz
NUC1 13C
P1 10.50 usec
PLW1 100.00000000 W
======== CHANNEL f2 ========
SFO2 600.1324005 MHz
NUC2 1H
CPDPRG[2 waltz16
PCPD2 70.00 usec
PLW2 25.00000000 W
PLW12 0.56250000 W
PLW13 0.28292999 W
F2 - Processing parameters
SI 32768
SF 150.9028085 MHz
WDW EM
SSB 0
LB 1.00 Hz
GB 0
PC 1.40
180
160
140
120
100
80
60
40
20
ppm

-

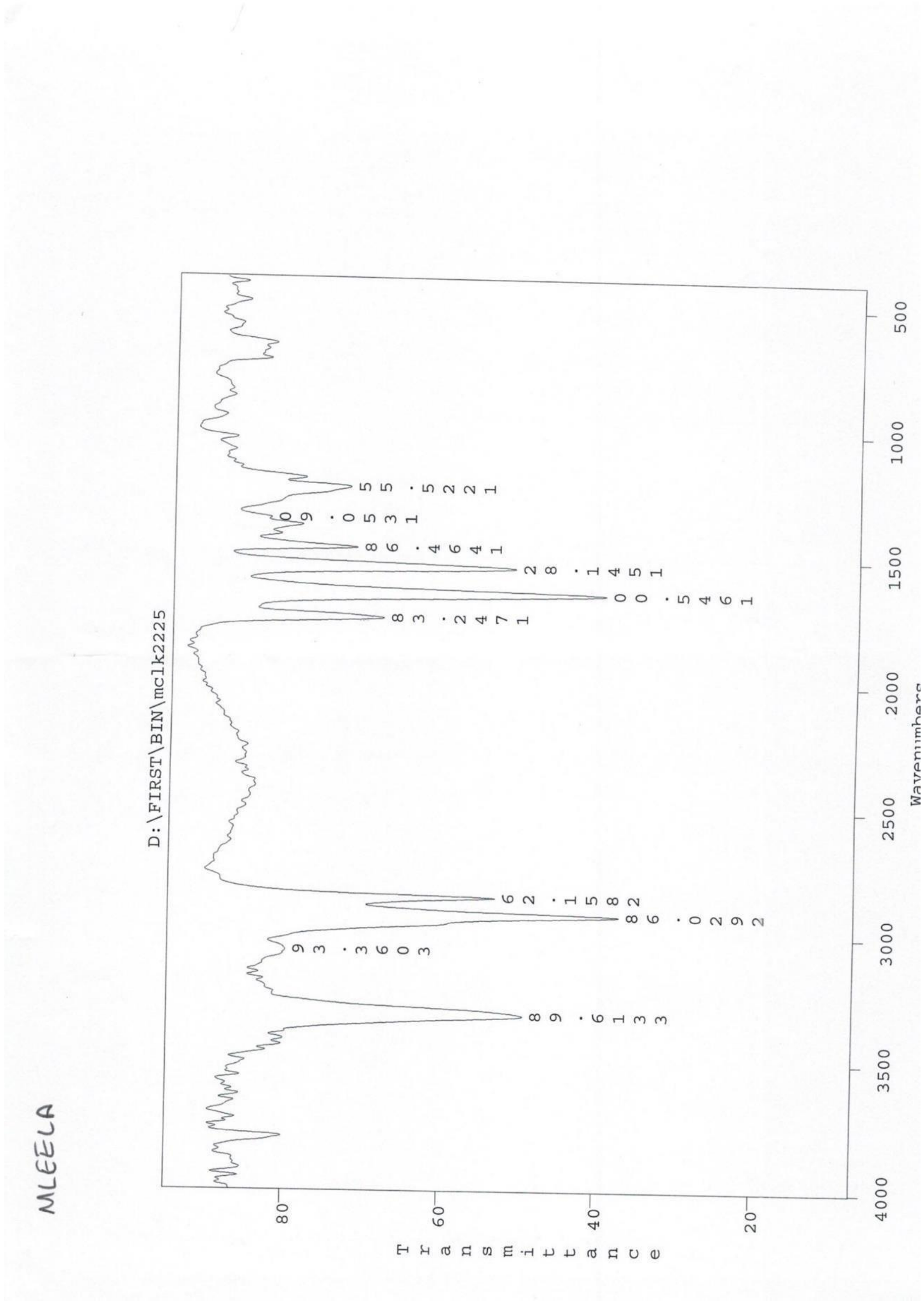
MLEELA
D:\FIRST\BIN\mclk2225
Transmittance
Wavenumbers
4000
3500
3000
2500
2000
1500
1000
500
80
60
40
20
3316.98
3063.39
2920.68
2851.26
1742.38
1645.00
1541.82
1464.68
1350.90
1225.55

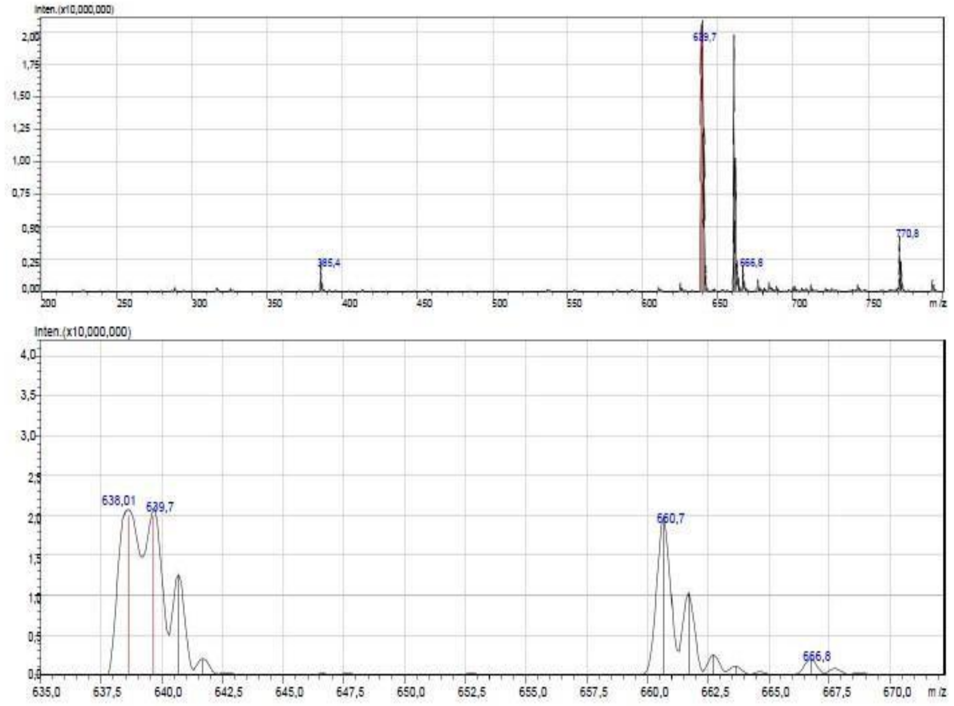

Inten.(x10,000,000)
639,7
385,4
666,8
770,8
m/z
Inten.(x10,000,000)
638,01
639,7
660,7
666,8
m/z

-
